# A structure-aware generative AI framework for revealing functional relationships in protein families

**DOI:** 10.1073/pnas.2536965123

**Published:** 2026-10-02

**Authors:** Divyanshu Shukla, Jonathan Martin, Faruck Morcos, Davit A. Potoyan

**Affiliations:** ^a^ Bioinformatics and Computational Biology Program, Iowa State University, Ames, IA 50011; ^b^ Department of Biological Sciences, The University of Texas at Dallas, Dallas, TX 75080; ^c^ Department of Bioengineering, The University of Texas at Dallas, Dallas, TX 75080; ^d^ Department of Physics, The University of Texas at Dallas, Dallas, TX 75080; ^e^ Center for Systems Biology, The University of Texas at Dallas, Dallas, TX 75080; ^f^ Department of Chemistry, Iowa State University, Ames, IA 50011; ^g^ Roy J. Carver Department of Biochemistry, Biophysics and Molecular Biology, Iowa State University, 50011, Ames, IA

**Keywords:** coevolution, protein structure, protein function, generative modeling, language models

## Abstract

Proteins can be studied through their sequence statistics or structural properties. These represent complementary views that are useful but lack a quantitative framework to tell, family by family, which is most informative and how to combine them. We introduce a framework that builds both views in parallel: amino acid (AA) alignments are translated into parallel alignments over a 3D interaction (3Di) structure-informed alphabet. Variational autoencoders compress each into a two-dimensional map, and direct coupling analysis places a shared coevolutionary energy on both maps, turning them into latent generative landscapes. On these landscapes, we define information-theoretic distance metrics that quantify how sequence changes drive structural and functional variation in protein families. We demonstrate the framework on five families: in malate dehydrogenases, the 3Di landscape identifies the structurally conserved scaffold that this family uses to encode thermal adaptation via sequence variability revealed in the AA landscape. In globins and transient receptor potential melastatin (TRPM), the 3Di landscape recovers known functional subfamilies. In the Flaviviridae E1 and E2 glycoproteins, structure reveals evolutionary relationships invisible at the sequence level. Because many sequences encode the same fold, our framework lets us disentangle family-sequence variability from structural and functional variation. These generative landscapes allow sampling near functional regions, and we show they can help us gain mechanistic insight into the evolutionary forces shaping sequence–structure–function variation and guide the design of new proteins.

## Significance

Protein databases contain millions of rapidly accumulating sequences, while increasingly accurate 3D structure predictions provide unprecedented data for analysis. Predicted structures can also be encoded using a structure-focused alphabet alongside standard amino acid (AA) representations. We introduce a framework that treats the AA and 3D interaction (3Di) alphabets as parallel views of a protein family, jointly navigated using a quantitative measure of coevolutionary fitness. By learning sequence landscapes defined by coevolutionary energies and quantifying their correspondence, we predict when AA and 3Di views overlap and when they provide independent information. Together, these representations yield a unified view of structural conservation and functional groupings across diverse protein families.

Proteins underpin a vast array of cellular functions, from energy metabolism to cell division. Deciphering their three-dimensional structures is key to understanding function, tracing evolutionary relationships, and guiding drug design (1–3). While protein sequence databases now contain hundreds of millions of entries, experimentally determined structures remain limited because traditional methods are time-consuming and costly. Recent advances in computational prediction, most notably AlphaFold2, have revolutionized structural biology by providing high-accuracy models at scale (4, 5). These models now support diverse applications, including structural alignment, pocket detection, complex modeling, novel fold discovery, and genome annotation refinement (6–8). Despite the success of sequence-based tools, remote homology detection remains a major bottleneck, leaving a substantial fraction of proteins functionally unannotated.

It is well understood that the sequence space is larger than the fold space, with multiple sequences encoding the same fold even at below 30% identity. A structure-aware representation can quantify how much of the variation in a collection of related sequences (family) is drift over a conserved fold. Furthermore, structure-based analysis can also provide a powerful alternative for identifying distant homologs and revealing functional and evolutionary relationships (9, 10).

The 3D interaction (3Di) alphabet encodes the spatial relationship between each residue and its nearest neighbor into one of 20 discrete geometric states, enabling scalable structure-based comparisons. Compared to classical structural alphabets, 3Di offers lower sequential dependency, more balanced state distributions, and higher information density localized within conserved structural cores (1, 11). ProstT5 builds upon this by fine-tuning the ProtT5 language model to translate bidirectionally between amino acid (AA) and 3Di sequences (12). This enables sensitive remote homology detection, rapid structure-aware searches, and de novo sequence generation from structural input (13).

These representations, however, still lack a way to organize an entire family into a single navigable picture. The latent generative landscape (LGL) framework addresses this for protein families at the AA level (14). The generative aspect of the landscape is built by using a variational autoencoder (VAE) (15) to compress a multiple sequence alignment (MSA) of a protein family into a two-dimensional latent space. Next, a Potts model inferred by direct coupling analysis (DCA) (16) on the same MSA assigns each decoded sequence a coevolutionary energy, which measures how well that sequence conforms to the family’s correlated-mutation patterns. The result is a navigable landscape where low-energy basins cluster functionally plausible sequences, and one can sample new candidate sequences near basins (14, 17).

**Fig. 1.**
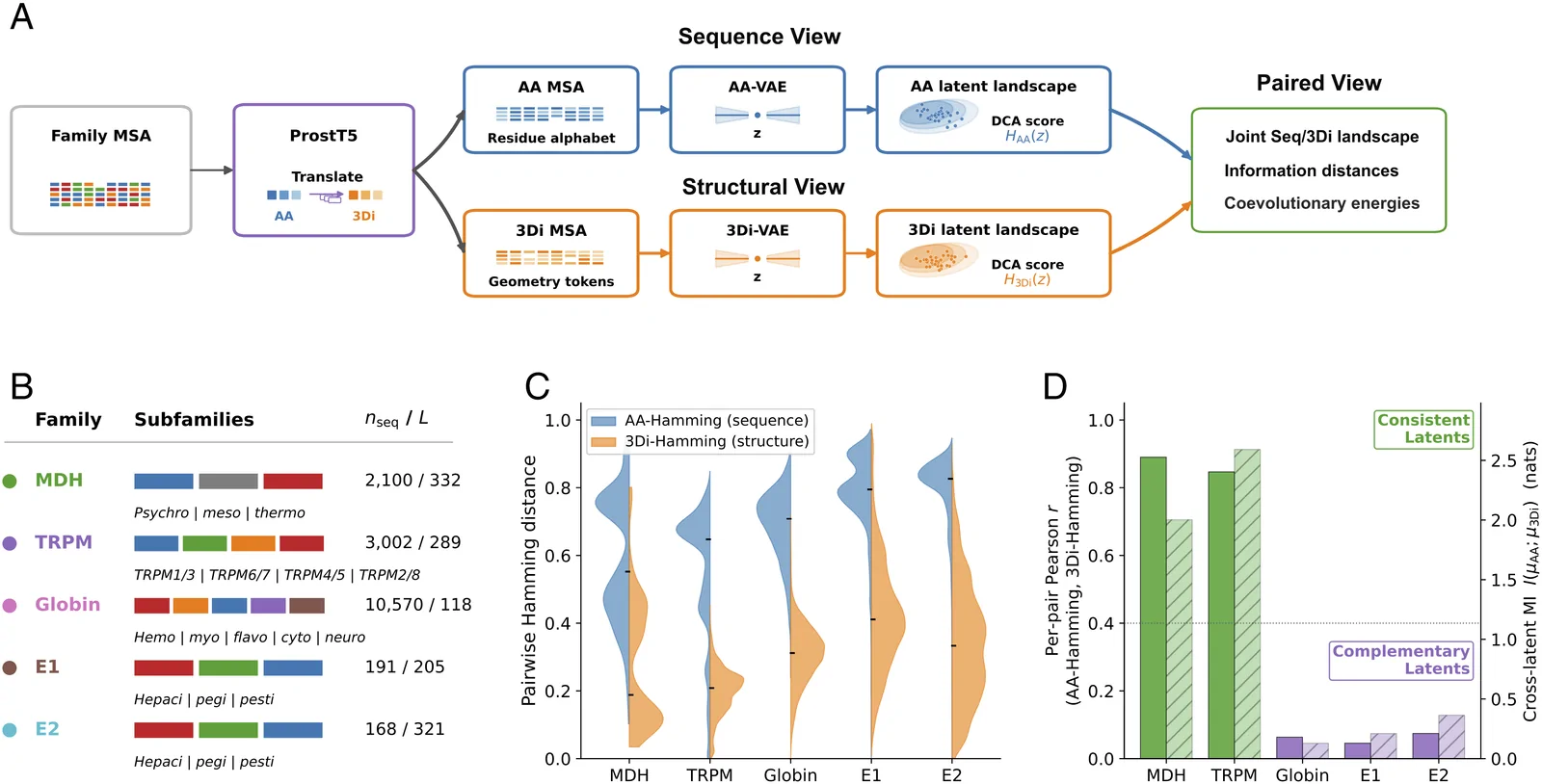
(*A*) Pipeline schematic. ProstT5 translates the family MSA into a 3Di structural-token alignment. The VAEs are then trained in parallel on the AA and 3Di alignments. The DCA on each alignment yields a Potts-model Hamiltonian on each latent. The two trained pipelines are then compared and/or combined. (*B*) The protein families studied, each row showing a family-color marker, a subfamily color strip with subfamily labels, and the sequence count and alignment length. (*C*) Per-family pairwise Hamming-distance distributions on the aligned MSAs, drawn as half-violins: blue on the left half shows AA-Hamming, orange on the right half shows 3Di-Hamming; short horizontal ticks mark the medians. (*D*) Per-family pairwise Pearson correlation between the AA-Hamming and 3Di-Hamming distance matrices on the left y-axis (solid bars), and cross-latent mutual information between the AA and 3Di encoders in nats on the right y-axis (hatched bars). Bars are colored by regime, green for the consistent latents and purple for the complementary latents; the dotted horizontal line on the left axis marks the regime threshold.

Here we build the same construction on the 3Di alphabet. We translate a family’s AA alignment into a parallel 3Di alignment, then pass it through the identical VAE-to-DCA pipeline. This yields a structure-token landscape, the 3Di-LGL, next to the conventional sequence landscape, the AA-LGL (Fig. 1*A*). Because the two are built from the same proteins by the same procedure and differ only in the alphabet, they can be read as parallel projections of one evolving family.

This parallel construction raises the question of when the two projections convey the same information, and when each carries information the other misses. We answer it with three metrics computed from the trained encoders. The metrics we use are the correlation between AA and 3Di pairwise distances, the mutual information between the two latent spaces, and a pullback Fisher-Rao metric that measures how sharply each decoder resolves sequence change at a given point. Across five families spanning a wide range of sequence and structural variation (Fig. 1*B*), these metrics separate a consistent regime, in which the 3Di-LGL largely restates the AA-LGL, from a complementary regime, in which it carries independent information. A family can be assigned to its regime from alignment statistics alone (Fig. 1*D*).

The two regimes prove biologically informative. In malate dehydrogenases (MDH), the 3Di-LGL holds a conserved scaffold nearly flat, while the AA-LGL separates psychrophilic from thermophilic cohorts, localizing thermal adaptation to sequence drift over a fixed fold. In globins and TRPMs, low-energy basins on the 3Di-LGL align with annotated functional subfamilies. In the Flaviviridae E1 and E2 glycoproteins, the structural view groups viruses that sequence identity leaves apart. Basin membership on the 3Di landscape further predicts simulated dynamics, and contact prediction from the two alphabets follows a bias–variance trade-off set by alignment depth. Together, these cases separate variation that reflects drift over a conserved fold from variation that changes structure and function, and they identify regions of each landscape where new sequences can be sampled.

## Results

Our framework represents each protein family as two parallel landscapes, one built from its AA sequences and one from its 3Di structural tokens, with a shared coevolutionary energy defined on both (Fig. 1*A*). Using ProstT5, we translate MSAs for each protein family into 3Di tokens that encode local residue geometry. We then process the 3Di MSAs with a VAE that maps sequences to latent coordinates and enables sampling across the latent space. Sequences decoded at each point were scored using a DCA-derived Potts-model Hamiltonian, yielding an energy landscape in which low-Hamiltonian regions correspond to structurally and functionally similar sequences.

Here, we extend prior LGLs studies to include the 3Di representation of sequences. In the LGLs, sequences in the same basin satisfy similar coevolutionary constraints. DCA energy organizes the latent space by structural and functional plausibility rather than by latent proximity alone. Due to the generative nature of the decoder, each landscape is not a static classification but a navigable map: one can sample latent coordinates near a functional basin and decode candidate sequences. Because the decoder is generative, each landscape is not a static classification but a navigable map: one can sample latent coordinates near a functional basin and decode candidate sequences that retain functional domain annotations and have the expected fold prediction (*Materials and Methods*).

### 3Di Reorganizes Structurally Similar but Sequence-Distant MDH Cohorts.

To examine how the AA and 3Di representations relate to structural constraints, we focus on the MDH family. In cytosolic MDHs from marine mollusks, psychrophilic and thermophilic (Fig. 1*B*) sequences occupy distinct regions in the AA latent space despite being known to retain similar overall structure, function, and dynamical behavior (17). Using the LGLs, we sampled two cohorts from separated regions of the AA landscape: one with 56 sequences from a psychrophilic-associated region (Cluster 1) and another with 43 sequences from a thermophilic/mesophilic region (Cluster 2).

At the MSA level, these two cohorts are sequence-distinct, with within-cohort pairwise identities of 40.9% and 47.3% and a between-cohort identity of 34.3% (see Fig. 1*C* for the MDH family). However, structurally they are nearly identical, having within- and between-cohort mean TM-score ≈0.92, well above the 0.5 homology threshold (18, 19).

This result is not specific to our cohort choice, as we find a similar pattern in a larger random sample of the family when the two clusters are defined purely by spectral clustering on the pairwise TM-score matrix rather than by the functional labels from Uniprot (*SI Appendix*, Fig. S1). The next question is whether the two latent representations register this grouping change.

**Fig. 2.**
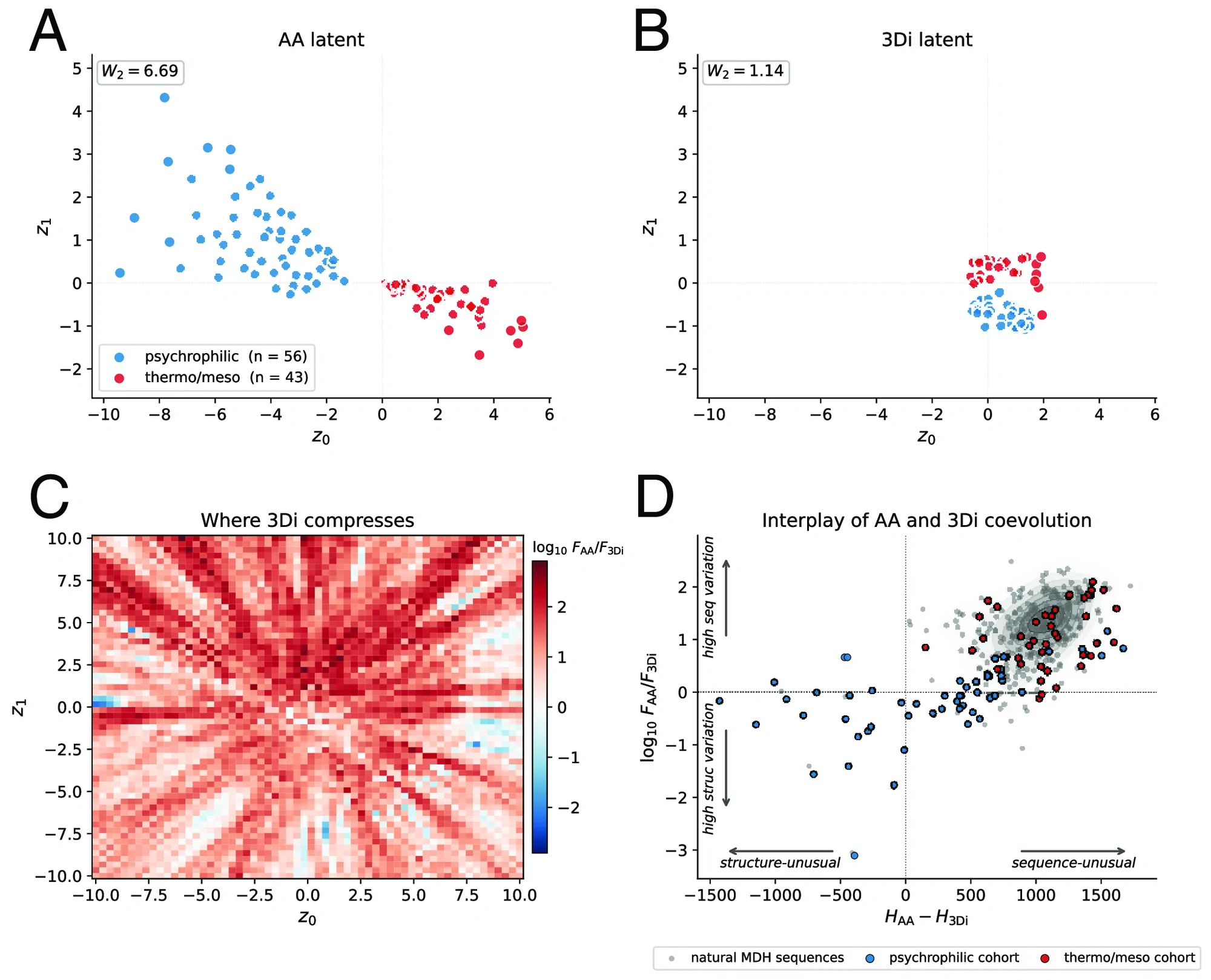
Building up sequence and structure-based latent representation of MDH (*A*) AA-LGL and (*B*) 3Di-LGL latent embeddings of the same two MDH cohorts (psychrophilic-associated and thermophilic-mesophilic), drawn on a common axis range for direct comparison. (*C*) Local AA-to-3Di Fisher determinant ratio across the MDH AA latent: red where the AA decoder is locally sharper, blue where the 3Di decoder is sharper. (*D*) Per-sequence AA-vs.-3Di plane. Horizontal axis: the AA-DCA minus 3Di-DCA Hamiltonian difference ΔH (*Right*: atypical to sequence coevolution but typical to structure); vertical axis: $\log_{10}\left(F_{\mathrm{AA}}/F_{3\mathrm{Di}}\right)$ (*Up*: the family diversifies faster in sequence than in structure). Gray, natural MDH sequences; blue and red, the psychrophilic and thermophilic-mesophilic cohorts.

The AA-LGL latent space encodes the sequence-level cohorts with a large between-cluster separation quantified by Wasserstein-2 distance (Fig. 2*A*). The 3Di-LGL latent space, by contrast, does not show this separation (Fig. 2*B*). The structural-token alphabet does not encode the cohort grouping change because the underlying structures are equally similar within and across cohorts. The Fisher-determinant ratio map (Fig. 2*C*) shows that the compression is heterogeneous, concentrated in the high-AA-sensitivity center. Because a different seed simply rotates the latent landscape, we checked whether the cohort-separation result was a seed artifact, as shown in Fig. 2*C*. A four-seed replicate of the AA-LGL and 3Di-LGL confirms that the within-vs.-between asymmetry in Fisher information holds across seeds in both alphabets (*SI Appendix*, Fig. S2). To compare the two latents at the sequence level, Fig. 2*D* plots two AA-vs-3Di derived quantities for each sequence: the difference of DCA Hamiltonians, $\Delta H=H_{\mathrm{AA}}-H_{3\mathrm{Di}}$, and the logarithm of the Fisher-determinant ratio, $\log_{10}\left(F_{\mathrm{AA}}/F_{3\mathrm{Di}}\right)$.

The horizontal axis separates sequences marked as atypical by the AA coevolutionary model (right) from those marked as atypical by the 3Di coevolutionary model (left). The vertical axis separates regions where the family diversifies in sequence faster than in structure (up) from those where structure diversifies faster (down).

The psychrophilic cohort sits near the origin, typical under both models. The thermophilic-mesophilic cohort lands in the upper-right quadrant: unusual to the AA model but typical to the 3Di one. That quadrant is also where the AA decoder has the larger Fisher determinant, and it is thus better able to distinguish between the two groups. The upper-right placement is mechanistically nontrivial: a gross structural change would have placed the cohort in the lower half, and a sequence-coevolutionary signal in an AA-poorly-resolved region would have placed it in the upper-left. The actual placement identifies thermal adaptation in MDH as fine-grained, sequence-level coevolution operating on a structurally conserved scaffold, a landscape-level analog of recent multiplexed hydrogen–deuterium exchange evidence that same-fold proteins exhibit substantial hidden variation in conformational fluctuation energies (20). Looking at the Fisher information of each latent space separately, instead of the ratio, clarifies that the AA latent space provides the cohort-distinguishing axis on its own, while the 3Di latent largely collapses the cohorts onto a common region (*SI Appendix*, Fig. S3).

Note that “psychrophilic” is an ecological label rather than a molecular one, so the few stragglers visible at higher ΔH likely correspond to cold-adapted MDHs that took divergent coevolutionary paths. The framework identifies them by their position in this AA-vs.-3Di plane. Together, the Hamiltonian and Fisher information are a useful diagnostic of the complementarity of the 3Di alphabet for specific protein sequences/cohorts, even though the MDH family overall sits in the consistent-latent regime (Fig. 1*D*), as explained in the following section.

**Fig. 3.**
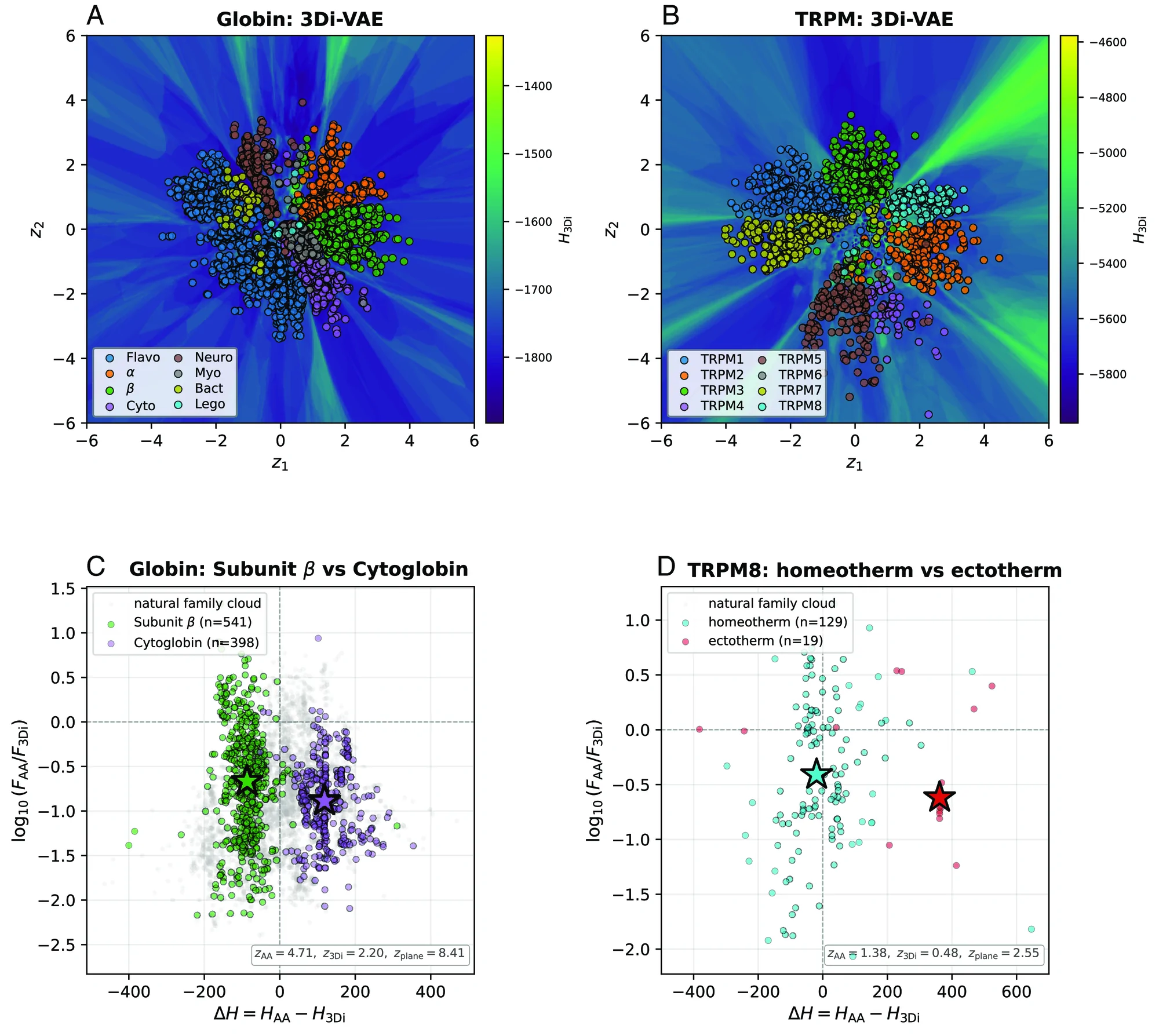
The 3Di-LGL clusters sequences into low-Hamiltonian basins corresponding to known functional subfamilies, and the AA-vs.-3Di plane resolves targeted within-family cohort experiments. (*A*) Globin family on the 3Di-LGL latent: background colormap shows the DCA Hamiltonian $H_{3\mathrm{Di}}$ (dark = low-*H* basin); overlaid training sequences are colored by UniProt-annotated subtype. (*B*) TRPM family on the 3Di-LGL latent, colored by channel subtype TRPM 1–8. (*C*) Globin Subunit *β* (green) vs. Cytoglobin (purple) on the AA-vs.-3Di plane. The two cohorts sit at AA-typical and AA-atypical positions of the plane despite sharing the same globin fold. Gray dots: full natural Globin cloud (recentered so ${\left\langle \Delta H\right\rangle }_{\mathrm{nat}}=0$). Stars: cohort medians. Stats *Inset*: cohort z-separation in AA latent, in 3Di latent, and on the plane. (*D*) TRPM8 homeotherm (cyan) vs. ectotherm (red) cohorts on the AA-vs.-3Di plane, partitioned by host-organism thermoregulation strategy. The *Insetz*-statistics in (*C* and *D*) report cohort separations in pooled-standard-deviation units: $z_{\mathrm{AA}}$ in the AA-LGL latent, $z_{3\mathrm{Di}}$ in the 3Di-LGL latent, and $z_{\mathrm{plane}}$ on the $\Delta H,\;\log_{10}F_{\mathrm{AA}}/F_{3\mathrm{Di}}$ plane itself.

The 3Di landscape thus reorganizes sequences by structural similarity, collapsing groups driven apart by sequence drift while preserving the larger barriers corresponding to function- or fold-level boundaries. Globin- and TRPM-family clustering by UniProt-annotated function and structural subdivision shows the same reorganization (Fig. 3) (21–23).

### Information Geometry Classifies AA/3Di Models as Consistent or Complementary.

Two multifamily analyses show that this latent space compression is a family-specific descriptor, not a universal feature. The per-family Fisher determinant ratio $F_{\mathrm{AA}}/F_{3\mathrm{Di}}$, when applied as an average over an entire landscape, measures the overall difference in how compressed the AA vs. 3Di representation is, but not necessarily whether the organization of the landscape has changed. MDH is the extreme AA-dominant case, Globin is strongly heterogeneous, TRPM and the Flaviviridae glycoproteins E1 and E2 all sit near parity, between 1.6 and 2.5× (*SI Appendix*, Fig. S4, with the spatial counterpart in *SI Appendix*, Fig. S5). Compression magnitude is therefore an independent descriptor that the regime classification does not predict: MDH and TRPM are both consistent-regime families yet differ fivefold in median compression.

The regime axis of Fig. 1*D* rests on two statistics. The first is model-free: how closely the AA-Hamming and 3Di-Hamming distances between pairs of sequences correlate to each other. The second is model-based: the cross-latent mutual information $I(\mu_{\mathrm{AA}};\;\mu_{3\mathrm{Di}})$, which measures how similar the groupings of sequences are between AA and 3Di landscapes. We estimate it from the trained encoders with the Kraskov–Stögbauer–Grassberger *k*-nearest-neighbor estimator (*SI Appendix*) and annotate the values on Fig. 1*D*. The two agree closely across families (Pearson r=0.97).

As a second, independent check, we used a geometric nearest-neighbor overlap measure, and it recovers the same consistent/complementary classification (Pearson r = 0.99; *SI Appendix*, Fig. S6). This agreement across three separate methods, including per-pair r, mutual information, and geometric overlap, gives confidence that the regime split reflects a real biological pattern rather than an artifact of any one metric. The underlying mathematical framework, a pullback Fisher-Rao geometry on the latent space, is described in *Materials and Methods* and expanded in *SI Appendix*, including per-family geodesics, a joint Fisher-Rao metric, and seed-robustness checks (*SI Appendix*, Figs. S2–S17).

### 3Di Landscapes Recover Known Functional Subfamilies and Resolve Within-Family Cohorts.

Across protein families, the 3Di-LGL clusters sequences into low-Hamiltonian basins that align with established functional annotations. In the globin family (Fig. 3*A*), basins of the landscape correspond to UniProt subfamily labels: cytoglobin, myoglobin, flavohemoglobin, neuroglobin, leghemoglobin, bacterial hemoglobin, and the hemoglobin *α*- and *β*-subunits each occupy distinct regions separated by Hamiltonian barriers (21).

The TRPM family shows the same pattern at the level of structural subdivision. Its eight channel subtypes (TRPM1–8) share gross domain architecture, differing in transmembrane and pore-loop details (22, 23). Even so, they separate into distinct basins on the 3Di landscape (Fig. 3*B*).

Globin sits in the complementary-latent regime (Fig. 1*D*) and TRPM in the consistent regime. Fig. 3 *A* and *B* show that the 3Di-LGL basins are directly interpretable as functional subfamilies without supervision in both regimes. We then test whether the Fisher ratio and Hamiltonian (Fig. 2*D*) are also connected to clustering changes between functional groups within the Globin and TRPM families.

For Globin, we contrast two proteins that share the same 8-helix fold but have very different functions (Fig. 3*C*). Subunit *β* is the blood-oxygen-transport hemoglobin chain, partnered in the $\alpha_{2}\beta_{2}$ tetramer. Cytoglobin is the ubiquitous cellular globin involved in Nitric oxide metabolism and cytoprotection. The two cohorts separate cleanly along $\Delta H=H_{\mathrm{AA}}-H_{3\mathrm{Di}}$, yet remain similarly placed in the 3Di latent space. Subunit *β* sits at AA-typical: its sequence stereotype dominates the hemoglobin-trained Potts model. Cytoglobin is shifted to AA-atypical: its free-standing cellular context diverges from those same sequence patterns. In TRPM8 (Fig. 3*D*), we partition the 150 natural TRPM8 sequences into homeotherm (mammals and birds, n=129) and ectotherm (reptiles, amphibians, and the coelacanth, n=19) cohorts. Despite identical channel-core fold (cohorts essentially indistinguishable in 3Di latent), the AA-vs.-3Di plane resolves the cohort split, with ectotherms shifted strongly to AA-atypical. The TRPM8 channel core has evolved sequence-level patterns adapted to environmental-temperature-variable physiology in ectotherms that the mammal-dominated Potts model registers as unusual, even though the fold is preserved. The clustering comparisons from both panels therefore demonstrate a sequence-vs.-structure asymmetry similar to Fig. 2*D*, providing evidence that the AA-vs.-3Di framework generalizes across both regimes (consistent and complementary) and across protein function classes (metabolic enzyme, oxygen-binding globin, transmembrane ion channel).

### Contact Prediction Follows a Bias–Variance Trade-Off Set by Alignment Depth.

**Fig. 4.**
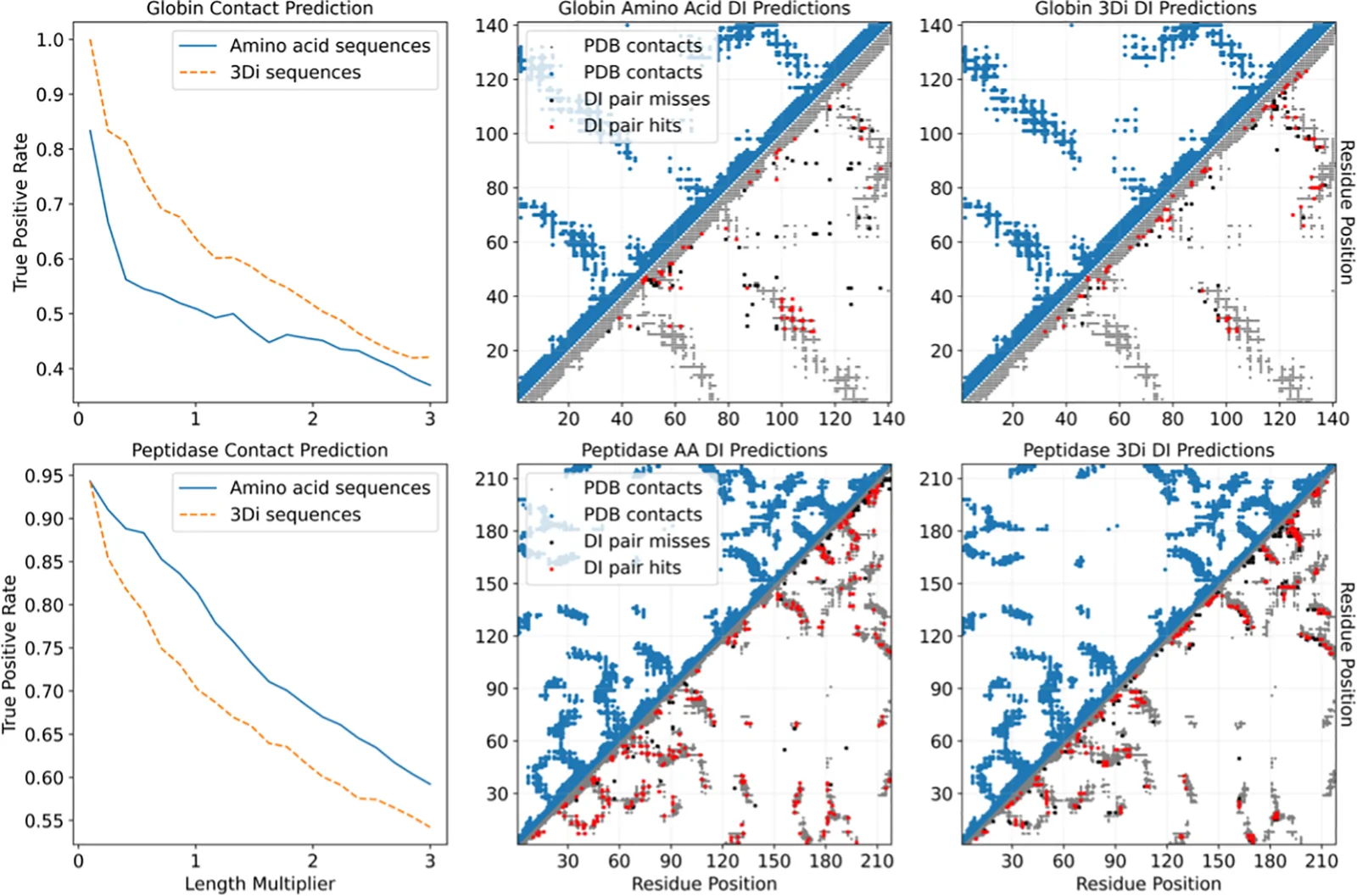
DCA contact prediction using 3Di and AA representations. Left column: true positive rate (TPR) of top-ranked direct-information pairs against PDB-derived Cβ–Cβ residue contacts (8 Å cutoff). Right column: corresponding predicted contact maps overlaid on the reference structures. Top row: globin family. Bottom row: cysteine peptidase family. For globin, the 3Di-MSA model yields a cleaner contact signal with fewer false positives; for peptidase, the AA-MSA model performs better. The reversal is consistent with a bias–variance trade-off between the two alphabets that depends on MSA depth (see also *SI Appendix*, Fig. S7 for subsampling analyses).

The complementarity between the AA and 3Di views also carries a practical consequence for a common use of a coevolutionary model, residue contact prediction, where it appears as a depth-dependent trade-off rather than a fixed winner. To evaluate the utility of 3Di representations for residue–residue contact prediction, we compared DCA performed on 3Di-based and AA-based MSAs for the globin and cysteine peptidase families. In the left column of Fig. 4, prediction performance is assessed by the true positive rate (TPR) of top-ranked direct information (DI) pairs against residue contacts derived from PDB structures. Because high-ranking DI pairs are well known to be enriched in structurally relevant contacts (16, 24, 25), this provides a natural benchmark for comparing the AA and 3Di alignments. In both families, DCA based on either representation yields reasonable to strong predictive power, but their relative performance differs by family. For globin, the 3Di MSA produces a clearer contact signal and fewer apparent false positives than the AA MSA, whereas for peptidase the AA representation performs better overall.

We interpret this difference as reflecting a bias–variance trade-off between the two encodings rather than the universal superiority of one representation over the other.

By compressing local structural environments into a reduced alphabet, 3Di lowers effective sequence entropy and the number of coupling parameters to estimate. This can reduce estimation variance and improve robustness when sequence depth is limited, or the family is highly divergent.

This appears to be the case for globin, where the smaller MSA means the AA model is more exposed to noise, and the reduced 3Di alphabet suppresses spurious couplings. In contrast, the peptidase family contains a much larger MSA (25,531 sequences vs. 10,381 for globin), allowing the richer AA alphabet to better exploit the available diversity and recover more informative couplings. In this regime, the reduced 3Di alphabet may dampen some of the fine-grained residue-level information that the AA representation retains. Consistent with this interpretation, subsampling analyses (*SI Appendix*, Fig. S7) show that 3Di generally provides better TPR at lower sequence counts, with the advantage becoming especially pronounced in the globin family. We therefore position 3Di as a complementary representation that is particularly advantageous in low-data or high-divergence settings, while AA representations remain preferable when large and diverse MSAs permit reliable estimation of the full residue-level coupling structure.

### Basin Membership on the 3Di Landscape Predicts Protein Dynamics.

**Fig. 5.**
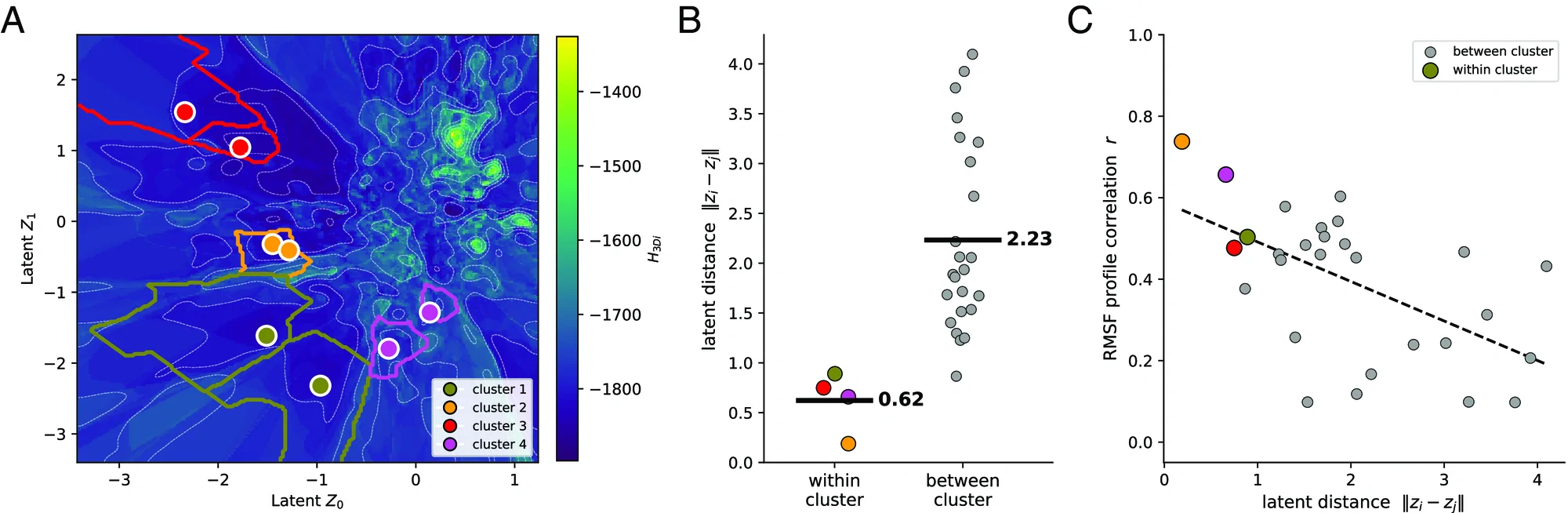
Dynamical similarity of flavohemoglobins tracks 3Di-LGL clustering. (*A*) Globin 3Di-LGL (zoom; background colormap is the Potts Hamiltonian $H_{3\mathrm{Di}}$, faint white lines are smoothed-landscape contours) showing eight representative flavohemoglobins drawn from four distinct low-Hamiltonian basins; basin outlines are the watershed of the smoothed landscape, and points are colored by cluster. (*B*) Within- vs. between-cluster Euclidean distance in the 3Di-LGL latent: same-basin representatives are ∼3.6× closer than different-basin representatives. (*C*) Per-residue RMSF profile correlation from 1.2 μs all-atom molecular dynamics (MD) vs. latent distance for all representative pairs.

Landscape clustering is useful only if its basins are biologically meaningful, not mere structural bookkeeping. Proteins that share similar folds and topological architecture often exhibit comparable dynamic behavior (26). Since the 3Di-based landscape clusters sequences into low-Hamiltonian basins, we tested quantitatively, rather than by visual inspection of the energy surface, whether basin membership predicts dynamical similarity. We selected eight representative flavohemoglobins from four distinct basins of the globin 3Di-LGL landscape (Fig. 5*A*), predicted their structures with AlphaFold2, and ran 1.2 μs all-atom MD for each. Representatives drawn from the same basin are markedly closer in the 3Di-LGL latent than those from different basins (Fig. 5*B*). We compared the resulting per-residue RMS fluctuation (RMSF) profiles across all pairs. Dynamical similarity decays significantly with latent distance (Fig. 5*C*): within-basin pairs share highly correlated fluctuation profiles. Between-basin pairs are systematically less correlated, though all are members of the same globin family. Basin membership on the 3Di-LGL therefore predicts MD, with the signal residing in the partition of the landscape into basins rather than in the visual height of the barriers between them.

### The 3Di View Reveals Flaviviridae Glycoprotein Relationships Invisible to Sequence.

The Flaviviridae E1 and E2 glycoproteins lie at the complementary-regime extreme of Fig. 1*D*, where the structural view carries evolutionary information that the sequence view does not. This makes them an ideal test of whether the 3Di landscape recovers relationships that the primary sequence misses. The Flaviviridae (27) is a highly diverse family of enveloped positive-sense RNA viruses that includes important pathogens of humans and other animals, as well as many viruses that pose emerging threats to human health (28–30). Previous reconstructions of Flaviviridae evolution have relied mainly on highly conserved proteins such as RNA-dependent RNA polymerase (RdRp). RdRp phylogeny supported the division of the Flaviviridae into three distinct clades: 1) an Orthoflavivirus/jingmenvirus group, 2) a clade comprising the large genome flaviviruses and members of the genus Pestivirus, and 3) a Pegivirus/Hepacivirus clade (*SI Appendix*, Fig. S8)(31, 32).

**Fig. 6.**
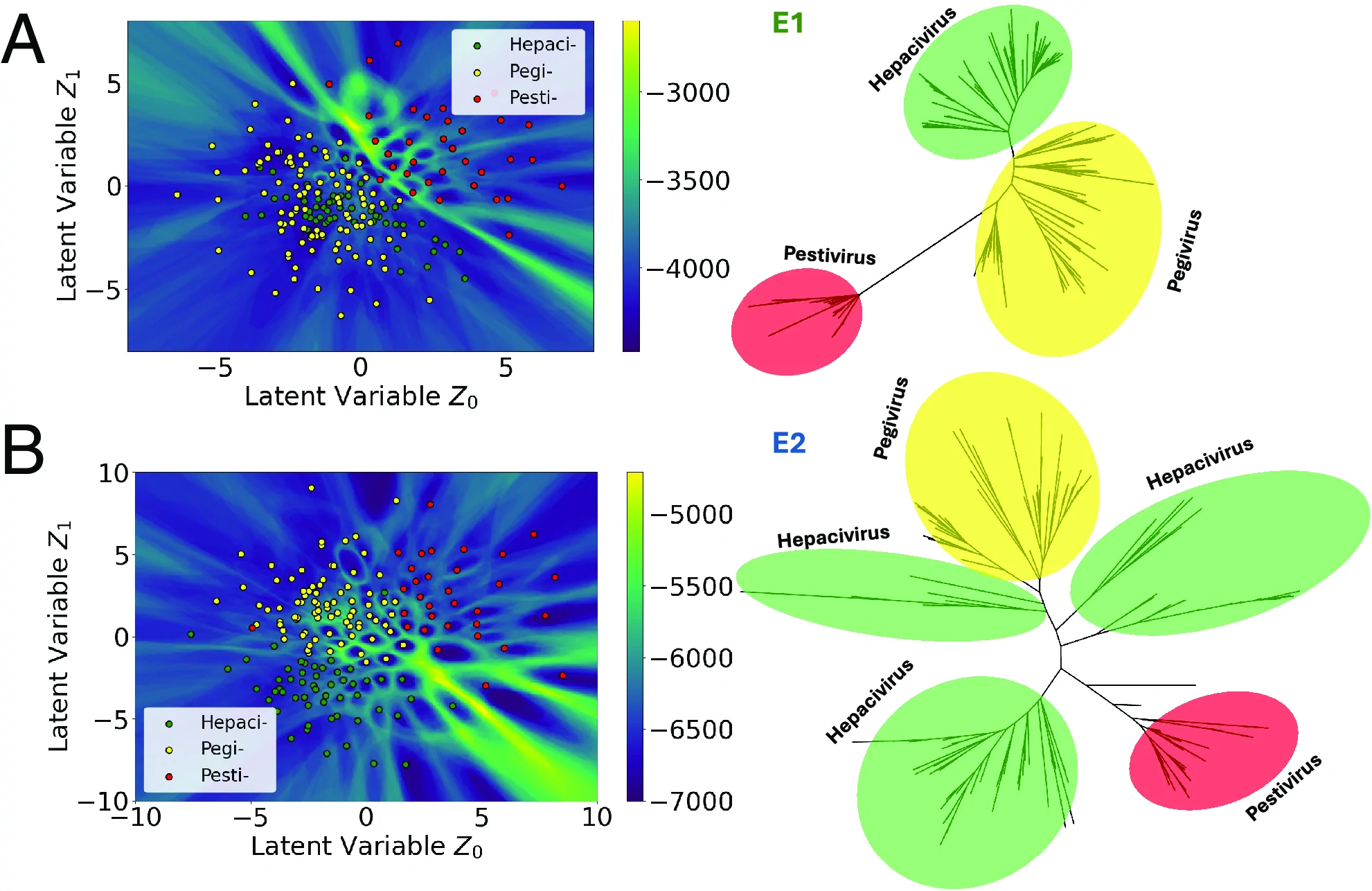
(*A*) *Left*, clustering of 3Di sequences in the landscape of E1 glycoprotein of the hepaciviruses, pegiviruses, and pestiviruses. *Right*, combined 3Di and AA-based E1 glycoprotein structural phylogeny. (*B*) *Left*, clustering of 3Di sequences in the landscape of E2 glycoprotein of the hepaciviruses, pegiviruses, and pestiviruses. *Right*, combined 3Di and AA-based E2 glycoprotein structural phylogeny.

These approaches provide limited resolution of glycoproteins, which are critical determinants of viral entry, host range, and immune recognition, due to high levels of sequence divergence. For example, E1 glycoproteins share only 10 to 15% AA sequence identity, while E2 glycoprotein identity ranges from 8.5 to 15% (33, 34). E1 and E2 glycoproteins of hepaciviruses, pegiviruses, and pestiviruses form a distinct class of fusion machinery and are strictly associated with vertebrate hosts (35, 36). Using 3Di sequences of structurally aligned E1 and E2 glycoproteins, we mapped their distribution in latent space and compared them with phylogenetic trees derived from the same proteins (Fig. 6). In the E1 (structurally conserved) landscape (Fig. 6*A*), pegiviruses and hepaciviruses strongly overlap, reflecting structural similarity that may be due to shared reliance on E1/E2 glycoproteins with internal ribosome entry site (IRES)–dependent translation (35). By contrast, pestiviruses cluster in a distinct region, suggesting a different structural orientation because they evolved from an ancestor with E glycoprotein and MTase, enabling cap-dependent translation (37). The E2 (structurally divergent) landscape (Fig. 6*B*) further underscores this division: pegiviruses and hepaciviruses remain closely positioned, while pestiviruses remain divergent.

One potential confounding factor is that small datasets tend to produce sparsely populated basins in the latent space. In our subsampling analysis, narrow point-centered wells are expected to be more frequent at lower sampling depth because the Potts-energy surface is estimated more coarsely under limited data (*SI Appendix*, Fig. S9). We observe that, even under changes in sampling conditions, basin-level and family-level organization is robustly preserved. Therefore, in the flaviviral glycoprotein landscapes, the meaningful signal is the consistent grouping of pegiviruses with hepaciviruses and the separation of pestiviruses.

In order to test the generality of this conclusion, we perform persistent-homology analysis of the AA-LGL and 3Di-LGL Hamiltonian landscapes in other families (*SI Appendix*, Fig. S10). More than 99% of raw local minima vanish under modest smoothing, while a small set of robust basins is consistently populated by training sequences. The persistence-ladder shape and the effective basin count differ between AA and 3Di in a family-dependent manner. This is a further indication that the relationship between the two representations is set family by family rather than universally.

The corresponding phylogenies (Fig. 6, *Right*) mirror these latent-space relationships, with pegiviruses and hepaciviruses forming tight clusters and pestiviruses branching separately. Together with RdRp phylogeny, these findings align with the theory that the gain of E1E2 evolution likely arose twice independently within the Flaviviridae: once in the Pegivirus/Hepacivirus clade and once in the Pestivirus lineage. In agreement with ref. 35, our findings also illustrate that evolutionary relationships among these highly divergent glycoproteins are largely inaccessible from primary sequence alone and become apparent only when structural information is incorporated (36).

## Discussion

A protein family could be analyzed in two ways: as a set of AA sequences, or as a set of folded structures. With recent advances in protein structure prediction, we can now represent the same family in a different alphabet, the 3Di alphabet, which records each residue’s geometry relative to its nearest neighbor as one of 20 discrete states. In some families the fold is preserved while sequences drift far apart. In others, sequence change may drive more substantial structural variation. Knowing which of these two phenomena is occurring can shed light on mechanisms of evolutionary adaptation and guide the design of new proteins.

In this work, we extend a generative framework to incorporate both representations. By using a VAE, we compress each alignment into its own two-dimensional map of the family, and use coevolutionary energies, inferred by DCA, to quantify the sequence probabilities for both alphabets. We then measure distances on these landscapes using Fisher information to determine whether these two representations are complementary. This lets us compare sequence and structural information, both visually through landscape analysis and through a set of model-based metrics. We found that by looking at the differences between these two representations we can conclude what is the main driver in functional diversification. In some cases, functional diversification is driven by changes at the AA level, while in others by conformational adaptation.

The central idea in our framework is how much these distinctions agree. When they agree closely, the consistent regime, the structural view is a remapping of the sequence view, and phylogeny and fold covary. When they do not, the complementary regime: structure forms a genuinely independent axis, conserved while sequence drifts. One example is the MDH family, where a structurally conserved scaffold has evolved toward stability at high temperatures through sequence variability. Conversely, for the Flaviviridae E1 and E2 glycoprotein families, a more accurate evolutionary tree can only be obtained by understanding structural changes across the family that are hidden in traditional phylogenetic analysis of their sequences. Our methodology reveals those distinctions.

Many sequences fold into the same structure, so a family is sampled far more densely in sequence space than in structure space. Across the five families studied in this work, the mean pairwise AA-Hamming divergence exceeds the mean 3Di-Hamming divergence by roughly twofold. This ratio reveals how redundant the sequence-to-structure map is.

Several limitations of our joint AA/3Di landscape framework remain. The 3Di alphabet is generally a compact representation of local structure and may miss longer-range structural dependencies that contribute to function. The latent landscapes, in turn, depend on the quality of the input MSAs and structural predictions. More fundamentally, 3Di-LGLs reproduce higher-order sequence correlations less faithfully than methods built expressly for that purpose (38). AA-LGLs, by contrast, can produce complex, in vitro functional sequences (39). Improving how the VAEs capture these correlations would strengthen its clustering and design power, especially where higher-order interactions matter.

Given the outlined limitations, a few directions follow naturally: conditioning the generative landscapes on functional features, adding physics-based scoring, or coupling them to MD to test the stability and dynamics of designed sequences. Recent large-scale experiments using multiplexed hydrogen–deuterium exchange mass spectrometry have measured conformational fluctuation energies across thousands of protein domains and found that proteins with the same fold and global stability can still hide substantial variation in their energy landscapes (20).

Our framework offers a computational angle to the same phenomenon by reorganizing sequences by local structural environment while preserving the larger barriers that separate functional groups. A natural test is whether the structural-diversity statistics obtained from an alignment predict the fluctuation magnitudes these experiments measure. Together, these extensions point toward structure-aware models that show how evolution has shaped a protein family and may help proteins with specific functional properties obtained from these LGLs.

## Materials and Methods

### MSA Acquisition and 3Di Sequence Generation.

For the analysis, we sourced MSAs from various origins. The MSA for MDH was obtained using the HMMSearch against the UniProt database, utilizing GREMLIN (40, 41). MSAs for globins and TRPM domains were referenced from the training dataset of sequence-based LGLs (14), and kinase MSAs from refs. 42 and 43. We saved all alignments in FASTA format. Prior to downstream modeling, we applied standard MSA filtering procedures to remove poorly aligned regions. Specifically, columns with a high fraction of gaps were excluded, and sequences with gap content >10% of sequence length were optionally filtered, so that the final alignment retained only the well-aligned core positions used for both VAE and DCA models. To translate these AA sequences into 3Di sequences, we employed ProstT5 (a state-of-the-art, pretrained protein language model) on three Nvidia A100 GPUs. ProstT5, an extension of ProtT5, encodes both sequence and structural information into 3Di tokens. The generated 3Di sequences were then reformatted to align with their respective AA MSAs, ensuring consistency for subsequent VAE analyses (11, 13).

### VAE Model Architecture.

The VAE (15, 44) models data *x* through a latent variable *z* with prior p(z), decoder $p_{\theta }\left(x|z\right)$, and an approximate posterior (encoder) $q_{\phi }\left(z|x\right)$. Training maximizes the evidence lower bound (ELBO):

[1] $$\begin{array}{c} \begin{array}{cc} \mathrm{ELBO}= & -E_{z∼q_{\phi }\left(z|x\right)}\log p_{\theta }\left(x|z\right)\\ & +D_{\mathrm{KL}}\left(q_{\phi }\left(z|x\right)\;\|\;p\left(z\right)\right), \end{array} \end{array}$$

where the first term is the reconstruction loss and the second regularizes each per-datapoint encoder posterior toward the standard-Gaussian prior. The encoder outputs the Gaussian parameters (μ,σ), and the reparameterization trick

[2] $$\begin{array}{c} z=\mu +\sigma ⊙\epsilon ,\;\epsilon ∼N(0,I), \end{array}$$

enables backpropagation through the stochastic sampling step. We use a two-dimensional latent ($z\in R^{2}$); the resulting aggregate posterior over the training set is shaped by the family’s data distribution rather than being Gaussian, and we map and analyze it throughout this paper.

### Data Representation and Decoding.

Sequences are represented as one-hot encoded vectors of dimensions q×L, where *L* is the sequence length and *q* is the alphabet size: q=20 for 3Di-LGL inputs and q=23 for AA-LGL inputs (the three additional symbols cover gap and noncanonical tokens). Each column contains a single one-hot entry indicating the symbol at that position. During decoding, the latent variables *z* are passed through a softmax activation to produce a probability distribution over the *q* symbols at each position; the output layer therefore has the same q×L dimensions as the input.

[3] $$\begin{array}{c} p{\left(a|z\right)}_{i}=\frac{\exp(\psi_{a_{i}}\left(z\right))}{\sum_{k\in A}\exp(\psi_{k_{i}}\left(z\right))}. \end{array}$$

This yields *L* rows with probability values summing to one. The reconstruction term in Eq. **1** vanishes when the decoded distribution at each position concentrates on the input symbol.

### Hyperparameters and Training.

All models were trained using 3 × L hidden units in both the encoder and decoder, where L is the input sequence length. We applied the ReLU activation function to all hidden layers. We used a latent dimensionality of 2. Optimization was performed using the Adam optimizer with a learning rate of $1\times 10^{-4}$, and L2 regularization with a penalty of $1\;\times \;10^{-4}$ was applied to the hidden units. We terminated training early if the loss did not improve for 50 consecutive epochs. Empirically, we observed that increasing the number of hidden units beyond 3 × L did not improve validation performance in the two-dimensional latent space. We implemented all models in TensorFlow (45) and trained them either on local workstations or on NVIDIA A100 GPUs in a high-performance computing cluster.

### Landscape Generation.

For each trained VAE model, we constructed a DCA (16) model using the same input sequences used to train the VAE. The DCA model defines the probability of a sequence S of length L based on observed statistics of AAs at individual positions $A_{i}$ and pairs of positions $\left(A_{i},A_{j}\right)$. This probability is given by

[4] $$\begin{array}{c} P\left(S\right)=\frac{1}{Z}\exp\left(\sum_{i<j}e_{\mathrm{ij}}\left(A_{i},A_{j}\right)+\sum_{i}h_{i}\left(A_{i}\right)\right). \end{array}$$

Here, $e_{\mathrm{ij}}$ represents pairwise coupling parameters between positions i and j, while $h_{i}$ corresponds to a local field term that captures the frequency of AAs at position i. This Potts form is the maximum-entropy distribution consistent with the observed one- and two-site marginals (44). These parameters characterize the Boltzmann-like distribution over sequences and can be inferred using various methods (46–48). To generate the landscape, we uniformly sampled coordinates $\left(z_{0},z_{1}\right)$ across the 2D latent space and passed them through the VAE decoder. The decoder produced a softmax probability distribution over the alphabet (Eq. **3**). From this, we extracted the maximum-probability sequence at each position as

[5] $$\begin{array}{c} H\left(S^{*}\right)=-\sum_{1\leq i\leq j\leq L}e_{\mathrm{ij}}\left(a_{i},a_{j}\right)-\sum_{i=1}^{L}h_{i}\left(a_{i}\right), \end{array}$$

where $S^{*}=a_{i}\ldots L$ and $a_{i}=\;\arg\;\max_{a\in A}\;p{\left(a|z\right)}_{i}$

Each decoded sequence was then scored using the Hamiltonian derived from the DCA model. This Hamiltonian reflects the sequence’s likelihood under the inferred coevolutionary constraints, enabling us to map evolutionary or structural plausibility across the latent space.

### Fisher Information Metric on the Latent.

The decoder of each trained VAE maps a latent point $z\in R^{2}$ to a probability distribution p(s|z) over sequences. The local distinguishability of nearby decoded distributions is measured by the Fisher information matrix in latent coordinates,

[6] $$\begin{array}{c} g_{\mathrm{ij}}\left(z\right)=E_{p(s|z)}\left[\frac{\partial \;\log\;p(s|z)}{\partial z_{i}}\;\frac{\partial \;\log\;p(s|z)}{\partial z_{j}}\right], \end{array}$$

which is the second-order approximation of the Kullback–Leibler divergence between decoded distributions,

[7] $$\begin{array}{c} \mathrm{KL}\left(p(s|z)\;\|\;p(s|z+\delta z)\right)=\frac{1}{2}\;\delta z^{⊤}g\left(z\right)\;\delta z+{O(|\delta z|}^{3}). \end{array}$$

Equivalently, g(z) is the Fisher–Rao metric on the simplex of categorical decoded distributions transported to the latent through the decoder Jacobian, following Arvanitidis et al. (49). We evaluate g(z) on a 60×60 latent grid by differentiating the decoder softmax with TensorFlow autograd.

The local Fisher determinant

[8] $$\begin{array}{c} F\left(z\right)=\sqrt{\det g(z)} \end{array}$$

is the volume element of the latent in units of nats of distinguishability per unit latent area. The per-family AA-to-3Di ratio $\log_{10}\left(F_{\mathrm{AA}}\left(z\right)/F_{3\mathrm{Di}}\left(z\right)\right)$ shown in Fig. 2 *C* and *D* and *SI Appendix*, Fig. S4 reports which decoder is locally sharper.

The Fisher–Rao geodesic length between two latent points $z_{a}$ and $z_{b}$ is the minimum value of

[9] $$\begin{array}{c} L\left[\gamma \right]=\int_{0}^{1}\sqrt{\dot{\gamma }{\left(t\right)}^{⊤}\;g\left(\gamma \left(t\right)\right)\;\dot{\gamma }\left(t\right)}\;dt, \end{array}$$

over smooth paths γ:[0,1]→Z with $\gamma \left(0\right)=z_{a}$, $\gamma \left(1\right)=z_{b}$. This length is the cumulative KL distinguishability of the decoded distributions along the optimal path and is the intrinsic distance on the latent space. We compute geodesics by minimizing the energy functional $E\left[\gamma \right]=\int_{0}^{1}{\dot{\gamma }}^{⊤}\;g\;\dot{\gamma }\;dt$ over cubic-spline parameterizations of *γ* with fixed endpoints. The joint AA + 3Di Fisher–Rao distance combines the two metrics block-diagonally as $L_{\mathrm{joint}}^{2}=L_{\mathrm{AA}}^{2}+L_{3\mathrm{Di}}^{2}$.

### Cohort-Separation Metrics.

Because the VAE latent has no intrinsic scale, we report cohort separations as scale-invariant quantities matched to each comparison. We use the ratio of Wasserstein-2 distances between cohort point clouds when comparing the same cohorts across the AA and 3Di latents (Fig. 2), a pooled-SD-standardized separation *z* when comparing across latents or derived planes of different scale (Fig. 3 *C* and *D*), and Euclidean latent distance when relating a per-sequence distance to an external observable, such as the RMSF correlations in Fig. 5, where a distribution-level Wasserstein measure does not apply. These three metrics are strongly correlated and rank cases the same way across all families and representations (*SI Appendix*, Fig. S11), so the distance-based conclusions are robust to the choice of metric. More fundamentally, the clustering does not require a distance metric at all: every pair of robust basins is separated by a Potts-energy barrier of 5 to $20\;\sigma_{H}$ (where $\sigma_{H}$ is the SD of the Potts Hamiltonian in the low-energy region, a model energy scale and not a physical temperature), so the landscape partitions sequences into energetically distinct basins as a topological property of the energy surface itself (*SI Appendix*, Fig. S12).

### Latent Space Entropy Calculation.

To assess the uncertainty of sequence generation across the latent space, we computed the entropy landscape using the decoder’s output distribution. For each coordinate in the latent space grid, the VAE decoder produces a Softmax distribution X over all AA symbols at every residue position. The average entropy per AA position at each coordinate measures variability or uncertainty in the generated sequences.

[10] $$\begin{array}{c} \hat{H}=-\frac{1}{L}\sum_{i=1}^{L}\sum_{q=1}^{20}P\;\left(x_{i}^{q}\right)\log\;\left(P\;\left(x_{i}^{q}\right)\right). \end{array}$$

Entropy landscapes computed this way are shown in *SI Appendix*, Fig. S13.

### AlphaFold2 Structure Prediction and Performance Evaluation.

To evaluate the structural relevance of sequences generated by the VAE, we used Colabfold (5, 50) to predict the 3D structures of both native and decoded sequences. We selected representative sequences from across the latent space grid and from regions of interest identified in the clustering and contact prediction analyses. We performed all predictions with no structural templates and five recycles.

We used the AlphaFold-generated structures in three core evaluations. First, to assess how well the latent space captures true structural similarity, we computed pairwise TM-scores between structures of clustered sequences. Second, to validate contact map predictions from DCA, we used AlphaFold structures as reference ground truth. Residue–residue contacts were defined based on a Cβ–Cβ distance cutoff of 8Å (Cα for glycine) and were further validated using dynamic contacts from MD simulations. Finally, we examined whether 3Di sequences with favorable DCA Hamiltonian scores also produced well-folded structures.

### MD Simulations.

We used the 3D structures predicted by AlphaFold2 as initial conformations for MD simulations, performed in OpenMM (51). We modeled proteins using the AMBER14-all force field, and represented water molecules with the TIP3P-FB model to construct fully solvated systems (52, 53). Each system was first energy-minimized, then equilibrated in the NVT ensemble (constant number of particles, volume, and temperature). This was then followed by a production run in the NPT ensemble (constant pressure and temperature) for a total duration of 1.2 μs at 298 K. To maintain thermodynamic stability, a Langevin Middle integrator was used to regulate the system temperature at 298 K, while pressure was controlled using OpenMM’s Monte Carlo barostat to maintain 1 atm pressure (54).

### Generation of Functional Sequences.

To generate protein sequences with specific functional characteristics, we utilized the trained VAE to decode latent coordinates into 3Di sequences (Fig. 1). We selected latent coordinates from regions of the landscape enriched for target domain annotations, based on their proximity to training sequences with known functions. The decoder produced 3Di token sequences at each sampled coordinate, which were subsequently translated into AA sequences using the ProstT5 decoder model. To prioritize structurally conserved sequences, we selected latent coordinates based on their DCA Hamiltonian values. We favored low-Hamiltonian regions to generate sequences hypothesized to retain native-like structure and functional domain features. The generated AA sequences were annotated using InterProScan (55) to assess the presence of conserved functional domains. For structural validation, we used AlphaFold2 to predict the 3D structures of selected generated sequences, and we assessed model confidence using per-residue pLDDT scores.

## Supplementary Material

Appendix 01 (PDF)

## Data Availability

The code used for training and generation of the results reported in this manuscript is available at https://github.com/PotoyanGroup/3Di-DCA-Landscape (56). All other data are included in the manuscript and/or *SI Appendix*.
