# Supplementary material for: A structure-aware generative AI framework for revealing functional relationships in protein families
Source: Proc Natl Acad Sci U S A. 2026 Oct 2;123(40):e2536965123. doi: 10.1073/pnas.2536965123 (PMC13643286; doi:10.1073/pnas.2536965123)
Supplement: Supplementary file 1 [file pnas.2536965123.sapp.pdf]

# Supporting Information for

## Supplementary Information for "A Structure-Aware Generative AI Framework for Revealing Functional Relationships in Protein Families"

**Divyanshu Shukla, Jonathan Martin, Faruck Morcos* and Davit A. Potoyan***

**Faruck Morcos, E-mail: faruckm@utdallas.edu; Davit A. Potoyan E-mail: potoyan@iastate.edu**

**This PDF file includes:**

Supporting text
Figs. S1 to S17

## Supporting Information Text

**Description of Supplemental Material.** This Supplementary Information collects the analyses that support the main-text framework but are not central to its narrative.

Supplementary Figs. S7–S9 present family-level robustness and reference analyses (MSA-size effect on contact prediction; the conserved RdRp landscape; landscape stability under MSA subsampling).

Supplementary Figs. S14–S15 and S4 quantify the cross-family information geometry that underpins the main-text claims: Supplementary Fig. S14 shows that Fisher-Rao geodesic distance recovers pairwise structural similarity far better than naive Euclidean distance on the 2D latents, justifying the Fisher-information framing introduced in the Cross-Family Information Geometry section; Supplementary Fig. S15 shows that the joint AA + 3Di Fisher-Rao distance is the best descriptor of pairwise similarity in the complementary-latent regime, while either single representation suffices in the consistent regime; Supplementary Fig. S4 reports the per-family Fisher determinant ratio $F_{\mathrm{AA}}/F_{\mathrm{3Di}}$, summarised at the median, the ninetieth percentile, and the integrated latent volume, and shows that the AA-over-3Di compression varies by family rather than being a universal constant.

Supplementary Figs. S3 and S13 provide single-family diagnostics: Supplementary Fig. S3 shows the single-latent Fisher-determinant-versus-Hamiltonian planes for the MDH AA and 3Di latents that the AA-versus-3Di plane of main-text Fig. 2 summarizes, and Supplementary Fig. S13 documents the decoder reconstruction-and-entropy analysis for the TRPM family, a family-specific result scoped accordingly in the main text.

Supplementary Fig. S10 establishes the topographic counterpart of the Fisher-geometry story: persistent-homology analysis of the Hamiltonian landscapes separates a small set of robust, training-sequence-populated basins from a long tail of finite-sampling noise minima, and shows that the persistence-ladder shape and the effective basin count differ between the AA and 3Di representations in a family-specific way.

Supplementary Fig. S1 extend the cohort transition picture across families, it summarizes the all-pairs Langevin barrier distributions per family and representation as violins ordered along the regime axis, with pooled medians and per-pair medians shown directly; barriers span roughly five sigma-natural-Hamiltonian units across families, with 3Di amplifying the cohort-to-cohort barrier in the consistent regime (MDH) and softening it in the complementary regime (Globin), while TRPM, intermediate between them, shows AA and 3Di nearly indistinguishable.

Supplementary Fig. S1 provides an independent validation of the within-versus-between cohort comparison in Fig. 2: a random sample of MDH AlphaFold structures is partitioned into two clusters by spectral clustering on the pairwise TM-score matrix itself, with no biological labels used; the data-driven clusters reach the same within-versus-between conclusion as the thermal-cohort study, confirming that the AA-versus-3Di asymmetry is robust to the choice of cluster boundary.

Supplementary Fig. S5 is the spatial counterpart of the per-family Fisher-ratio summary in Supplementary Fig. S4: for each of the five families, it shows where in latent space the AA decoder is locally sharper than the 3Di decoder (top row) and where the local Fisher metric collapses toward a near-one-dimensional corridor in either representation (bottom row); these maps make the family-level differences in AA-versus-3Di compression and anisotropy directly visible.

Supplementary Fig. S6 documents the full information-bottleneck framework that underpins the regime classification cited throughout the paper: the IB plane visualizing the AA-versus-3Di shift for each family, the cross-latent mutual information per family, the predictive law that links cross-latent mutual information to the model-free structural-diversity statistic of the multiple sequence alignment, and the cross-metric validation showing that an entirely independent geometric metric recovers the same regime classification. Together, these results provide the geometric basis for the main-text recommendation to interpret latent distances via Fisher-Rao rather than Euclidean geometry, and to combine the AA and 3Di latents for complementary-regime families.

Supplementary Figs. S11–S12 close the loop on the latent-distance question from two complementary directions: Supplementary Fig. S11 shows that the three latent-distance metrics (Euclidean, Fisher-Rao geodesic, and Wasserstein-2) capture the same separation trends across all families and representations (Spearman $\rho = 0.92$ to $0.99$), so the distance-based conclusions do not depend on the choice of metric; and Supplementary Fig. S12 shows that the result does not require a distance metric at all, since every pair of robust basins is separated by a Potts-energy barrier of 5 to $20\,\sigma_H$ (a model energy scale, not a physical temperature), so the landscape partitions sequences into energetically distinct basins as a topological property of the energy surface.

Finally, Supplementary Figs. S2–S17 document a seed-replicate robustness control on the MDH cohort study: Supplementary Fig. S2 shows that retraining the AA-VAE and the 3Di-VAE under four independent random seeds reproduces the within-versus-between Fisher-Rao contrast (cohort-separation ratio $\approx 5.6$ for both alphabets, CV $\approx$ 13–14% across seeds) even though the absolute latent picture rotates seed-to-seed, while Supplementary Fig. S17 reports the sequence-anchored counterpart and shows that the per-sequence local AA/3Di Fisher ratio is reproducibly positive for the psychrophilic cohort but sign-dependent on the seed for the thermo/mesophilic cohort, so the within-versus-between cohort contrast (not the per-pixel local Fisher value) is the seed-robust load-bearing claim.

**Definitions of quantitative measures.** This section states the exact computation behind each quantitative measure used in the supplementary figures, so that every reported number is unambiguous. Throughout, $N$ is the number of training sequences in a family, and $\mu^{(i)}_{\mathrm{AA}}, \mu^{(i)}_{\mathrm{3Di}} \in \mathbb{R}^2$ are the encoder posterior means (latent coordinates) of sequence $i$ in the AA-VAE and 3Di-VAE, respectively.

**Cross-latent mutual information (Fig. S6).** The cross-latent mutual information is the entropy of one latent embedding minus the entropy that remains once the other is known, written as the difference between the summed marginal entropies and the joint entropy of the paired latent coordinates,

$$I(\mu_{\rm AA};\mu_{\rm 3Di}) = S(\mu_{\rm AA}) + S(\mu_{\rm 3Di}) - S(\mu_{\rm AA},\mu_{\rm 3Di}), \qquad [1]$$

where $S(\,\cdot\,) = -\int p\,\ln p$ is the differential (Gibbs–Shannon) entropy in nats. Equivalently, $I$ is the relative entropy between the joint distribution and the product of marginals, $I = \big\langle \ln\big[\,p(\mu_{\rm AA},\mu_{\rm 3Di})/p(\mu_{\rm AA})p(\mu_{\rm 3Di})\,\big]\big\rangle$, so $I = 0$ iff the two embeddings are statistically independent and $I > 0$ measures how much the structural and sequence latents constrain each other. We evaluate the entropies nonparametrically with the Kraskov–Stögbauer–Grassberger (KSG) $k$-nearest-neighbor estimator (1) ($k = 5$), which makes no Gaussian or variance assumption: $\hat{I} = \psi(k) + \psi(N) - \langle \psi(n_x^{(i)}{+}1) + \psi(n_y^{(i)}{+}1)\rangle_i$, with $\psi$ the digamma function and $n_x^{(i)}, n_y^{(i)}$ the marginal neighbor counts within the joint $k$-th-neighbor Chebyshev radius of point $i$. Values are reported in nats with bootstrap confidence intervals.

**$k$-nearest-neighbor overlap (Fig. S6).** For each sequence $i$, let $\mathcal{N}_k^{\rm AA}(i)$ and $\mathcal{N}_k^{\rm 3Di}(i)$ be its $k$ nearest neighbors (excluding itself) under the Euclidean metric of the AA and 3Di latents. The per-sequence overlap is

$$O_k(i) = \frac{\big|\,\mathcal{N}_k^{\rm AA}(i)\cap\mathcal{N}_k^{\rm 3Di}(i)\,\big|}{k}, \qquad [2]$$

and the family-level value is $\langle O_k\rangle_i$ (we report $k = 10$).

**Per-family Fisher determinant ratio (Fig. S4).** Let $F(z) = \sqrt{\det g(z)}$ be the Fisher determinant (main-text Methods), evaluated on the shared latent grid. The per-family summary ratios in Fig. S4 are *ratios of grid-wide summary statistics of each map*, not the summary of a pointwise ratio:

$$R_{\rm med} = \frac{{\rm median}_z\,F_{\rm AA}(z)}{{\rm median}_z\,F_{\rm 3Di}(z)}, \qquad R_{\rm P90} = \frac{Q_{90}[F_{\rm AA}]}{Q_{90}[F_{\rm 3Di}]}, \qquad R_{\rm tot} = \frac{\sum_z F_{\rm AA}(z)}{\sum_z F_{\rm 3Di}(z)}, \qquad [3]$$

where $Q_{90}$ is the ninetieth percentile over grid points and $R_{\rm tot}$ is the ratio of integrated latent volumes. By contrast, the *maps* in main-text Fig. 2C and Fig. S5 show the *pointwise* log ratio $\log_{10}\!\big(F_{\rm AA}(z)/F_{\rm 3Di}(z)\big)$ at each grid point; the two presentations answer different questions (where compression happens versus how much it amounts to family-wide).

**Basin persistence, robust basins, and effective basin count (Fig. S10).** Basins are not hand-picked. On the $500\times500$ Hamiltonian grid $H(z)$ we apply a Gaussian smoothing ($\sigma = 1$ grid unit), detect *all* local minima, and watershed-segment the smoothed surface seeded by those minima, so every grid point is assigned to a basin. For each basin $b$ with minimum height $H_b^{\rm min}$, its persistence is

$$p_b = s_b - H_b^{\rm min}, \qquad [4]$$

where $s_b$ is the saddle height at which basin $b$ first merges into a deeper basin (obtained by union-find on the watershed adjacency graph under a sublevel-set filtration). Basins are ranked by $p_b$; the "robust" set is the top $K = 4$. The effective number of basins reported in Fig. S10D is the participation ratio of the persistences,

$$K_{\rm eff} = \frac{\big(\sum_b p_b\big)^2}{\sum_b p_b^2}, \qquad [5]$$

which equals the true basin count when all basins are equally persistent and falls toward 1 when one basin dominates. (We use the participation ratio; note this is the effective count, not its reciprocal.) The occupancy-based count $K_{\rm seq\,eff}$ is the same formula applied to the number of training sequences assigned to each basin.

**Inter/intra basin separation ratio (Fig. S11).** For a set of labeled groups in a given latent and metric, let $D_{ab}$ be the mean pairwise distance between groups $a$ and $b$. The separation ratio is

$$\rho_{\rm sep} = \frac{\overline{D_{ab}}\ (a\neq b)}{\overline{D_{aa}}}, \qquad [6]$$

the mean inter-group (between-basin) distance divided by the mean intra-group (within-basin) distance; values $> 1$ indicate groups that are more separated than they are internally spread. This is computed identically for the Euclidean, Fisher-Rao geodesic, and Wasserstein-2 metrics.

**Langevin cohort barriers and energy normalization (Figs. S16 and S12).** Two distinct Hamiltonian-spread normalizations appear and should not be conflated. For the Langevin barriers (Fig. S16), $\sigma_{H,\rm nat}$ is the standard deviation of the Potts Hamiltonian evaluated at the latent positions of the natural training sequences. A per-trajectory barrier is the maximum smoothed Hamiltonian reached along the productive crossing segment minus the source-basin floor, expressed in units of $\sigma_{H,\rm nat}$. For the metric-free saddle barriers (Fig. S12), $\sigma_H$ is instead the standard deviation of the Potts Hamiltonian in the low-energy region of the landscape, and the barrier between two basins is the minimax (lowest-saddle) value along the minimum-energy path, in units of $\sigma_H$. Both $\sigma$ scales are internal model energy scales, not physical temperatures.

**Information geometry: pullback Fisher metric, Fisher-Rao geodesics, and regime classification.** The pullback Fisher information metric defined in the main-text Methods, $g_{ij}(z) = \mathbb{E}_{p(s|z)}[\partial_i \log p(s|z)\, \partial_j \log p(s|z)]$, makes the latent space a Riemannian manifold whose geodesics minimize the cumulative Kullback–Leibler divergence between decoded sequence distributions (2, 3). The same Fisher-information structure underlies the thermodynamic-length framework for finite-time stochastic protocols, in which the Fisher-Rao length of a path lower-bounds the excess work required to traverse it (4); in our setting, this length is the minimum cumulative distinguishability of decoded sequence distributions along any path connecting two latent points. Geodesics are computed by minimizing the energy functional $E[\gamma] = \int_0^1 \dot{\gamma}^\top g\, \dot{\gamma}\, dt$ over cubic-spline paths with fixed endpoints at pairs of training-sequence encodings.

With this Fisher-Rao geodesic, the 3Di latent recovers pairwise structural similarity (3Di-Hamming distance) in every family at Pearson $r = 0.25$–$0.63$, consistently improving on naive Euclidean distance (Supplementary Fig. S14A). The AA latent recovers structural similarity only in the consistent regime (MDH, TRPM); for the complementary-regime families (Globin, E1, E2) the AA latent is essentially uninformative about structure (Supplementary Fig. S14B). When the two views are combined into the joint Fisher-Rao distance, $L^2_{\text{joint}} = L^2_{\text{AA}} + L^2_{\text{3Di}}$, the joint metric is the strongest predictor of pairwise similarity in the complementary regime(Supplementary Fig. S15). Together with the per-family Fisher determinant ratio of Supplementary Fig. S4, the spatial maps of Supplementary Fig. S5, and the cross-latent mutual information and k-nearest-neighbor overlap of Supplementary Fig. S6, this gives the usable recipe stated in the main text: classify a family by its model-free per-pair $r$ (Fig. 7D), then either pick the sharper Fisher field (consistent regime) or combine both via the joint Fisher-Rao metric (complementary regime).

**Seed-replicate robustness control.** To check that these conclusions do not depend on the specific VAE training run, we retrained the MDH AA-VAE and the MDH 3Di-VAE under four independent random seeds (same MSA, same architecture, 50 epochs each), recomputed the pullback Fisher metric, re-encoded the psychrophilic and thermo/mesophilic cohorts under each seed, and compared within-cohort versus between-cohort Fisher-Rao distances on 400 sampled real sequence pairs per seed (Supplementary Fig. S2). The 2D latent picture visibly rotates and reflects across seeds in both alphabets, consistent with the residual orthogonal gauge that the standard-normal prior leaves unconstrained. In every seed, however, the between-cohort Fisher-Rao distance is several-fold larger than the within-cohort distance, and the cohort-separation ratio (median between / mean within) is $5.64 \pm 0.79$ for AA and $5.57 \pm 0.74$ for 3Di across the four seeds (CV $\approx$ 13–14%). The two alphabets therefore agree on the cohort-separation magnitude, and the within-versus-between contrast is the seed-robust load-bearing quantity rather than the absolute Fisher magnitude.

By contrast, the local per-sequence AA/3Di Fisher ratio is more gauge-dependent (Supplementary Fig. S17). Its median is reproducibly positive for the psychrophilic cohort across all four seeds ($+0.68$, $+0.43$, $+0.60$, $+0.46$ in $\log_{10}$ units, mean $+0.54 \pm 0.12$): the AA decoder is consistently locally sharper at the psychrophilic sequence locations. The median for the thermo/mesophilic cohort, however, spans 0.56 dex across the four seeds (from $+0.20$ in seed 0 to $-0.36$ in seed 3): the sign of the local AA-versus-3Di ratio at this cohort is not strictly seed-robust. Main-text Fig. 2C is therefore an illustrative single-seed view of the local compression pattern; the quantitatively load-bearing claim about cohort separation is the seed-robust within-versus-between Fisher-Rao contrast of Supplementary Fig. S2.

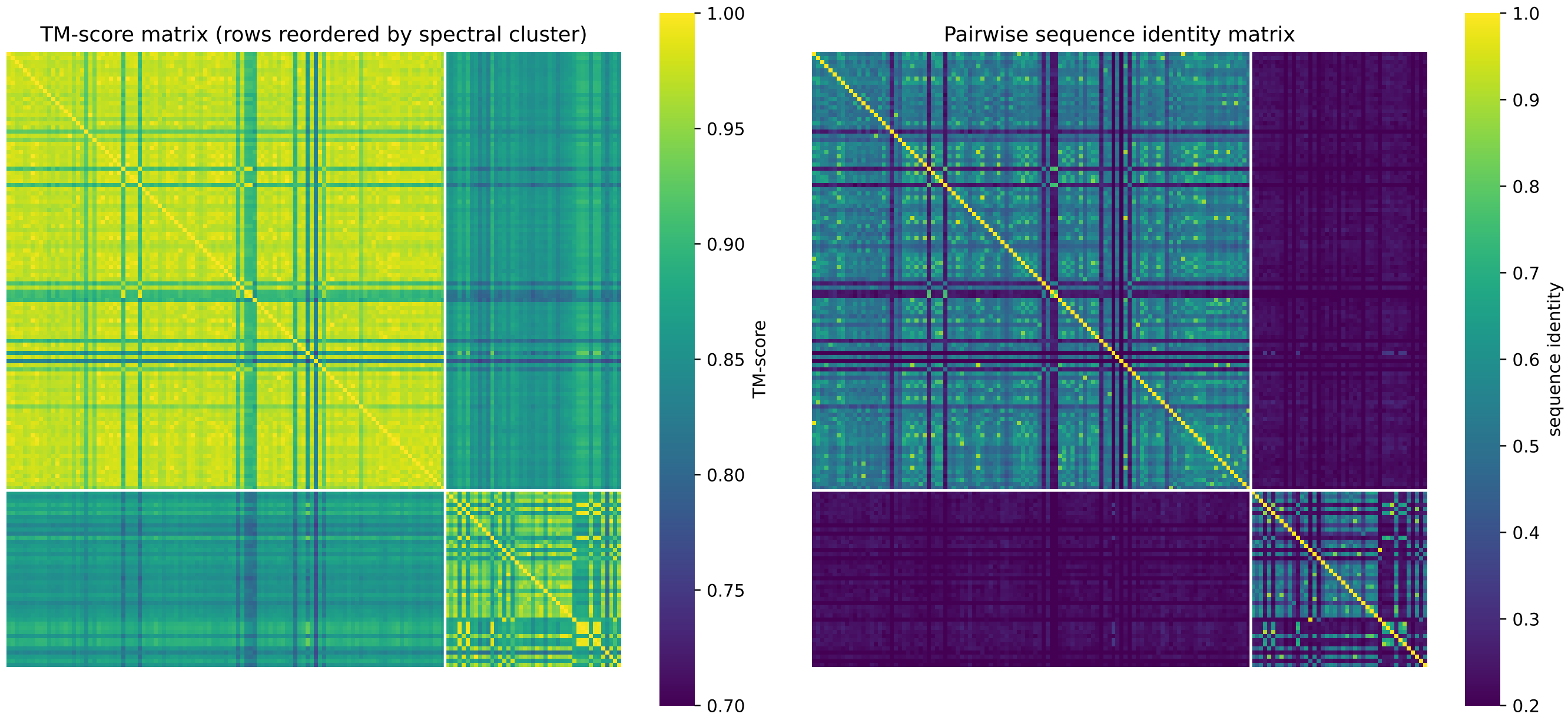


**Fig. S1.** Within-versus-between cohort asymmetry on the MDH family under purely structural clustering, an independent validation of the cohort analysis in main-text Fig. 2. A random sample of MDH AlphaFold structures is subjected to all-versus-all TM-align, and the resulting pairwise TM-score matrix is partitioned into two clusters by spectral clustering on the TM matrix alone, with no biological labels used. **(Left)** Pairwise TM-score matrix reordered by spectral cluster. The within-cluster blocks along the diagonal are brighter than the between-cluster off-diagonal block, but the contrast is modest — structures are similar throughout the sample. **(Right)** Pairwise sequence-identity matrix reordered identically. The between-cluster block shows a much more pronounced drop in identity than the corresponding TM-score block, illustrating that sequence space magnifies cluster separation relative to structure space across the MDH family. The data-driven, label-free clustering reaches the same within-versus-between conclusion as the thermal-cohort study in Fig. 2, confirming that the AA-versus-3Di compression result is not an artifact of the thermal-cohort labeling.

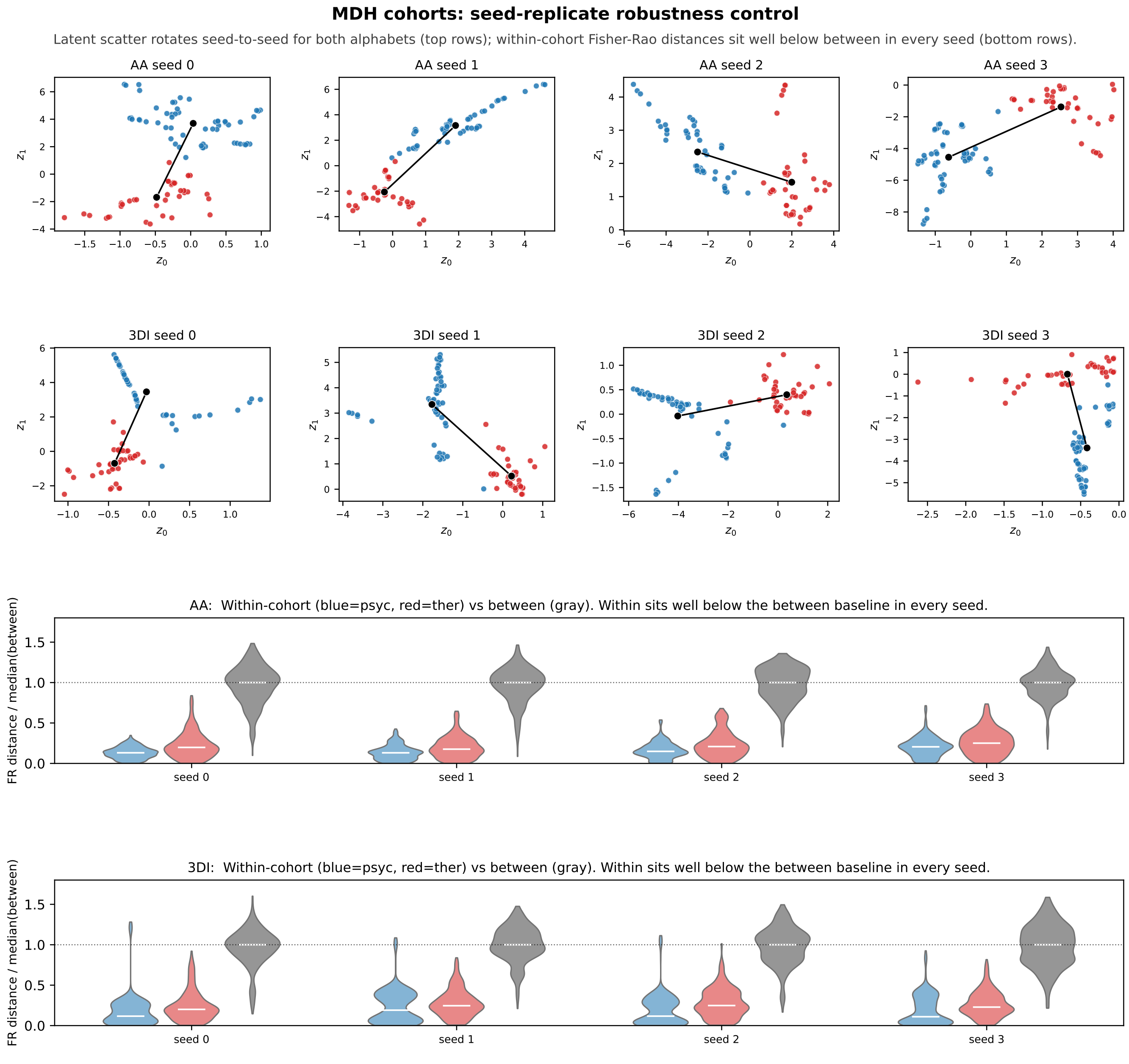


**Fig. S2.** Seed-replicate robustness control on the MDH cohorts. The MDH AA-VAE and 3Di-VAE were each retrained under four independent random seeds (same MSA, same architecture, $50$ epochs each). **Rows 1–2:** Latent embeddings of the psychrophilic (blue) and thermo/mesophilic (red) cohorts under each seed for the AA latent (row 1) and the 3Di latent (row 2), with the cohort-centroid line drawn in black. The cohort cloud visibly rotates and reflects across seeds in both alphabets, consistent with the residual orthogonal gauge that the standard-normal prior pins. **Rows 3–4:** Within- versus between-cohort Fisher-Rao distance distributions on $400$ sampled real sequence pairs per seed, normalized by that seed's median between-cohort distance (dashed line at unity). Within-cohort distances (psyc=blue, ther=red) sit at $\sim 0.1$–$0.3$ of the between-cohort baseline (gray) in every seed and both alphabets. The cohort-separation ratio (median between $/$ mean within) is $5.64 \pm 0.79$ for AA and $5.57 \pm 0.74$ for 3Di across the four seeds (CV $\approx 13$–$14\%$). The within-versus-between contrast is therefore seed-robust, while the absolute latent picture is not. Underlying scripts: `73_train_mdh_seeds.py`, `74_seed_fisher_distances.py`, `75_seed_replicate_figure.py`.

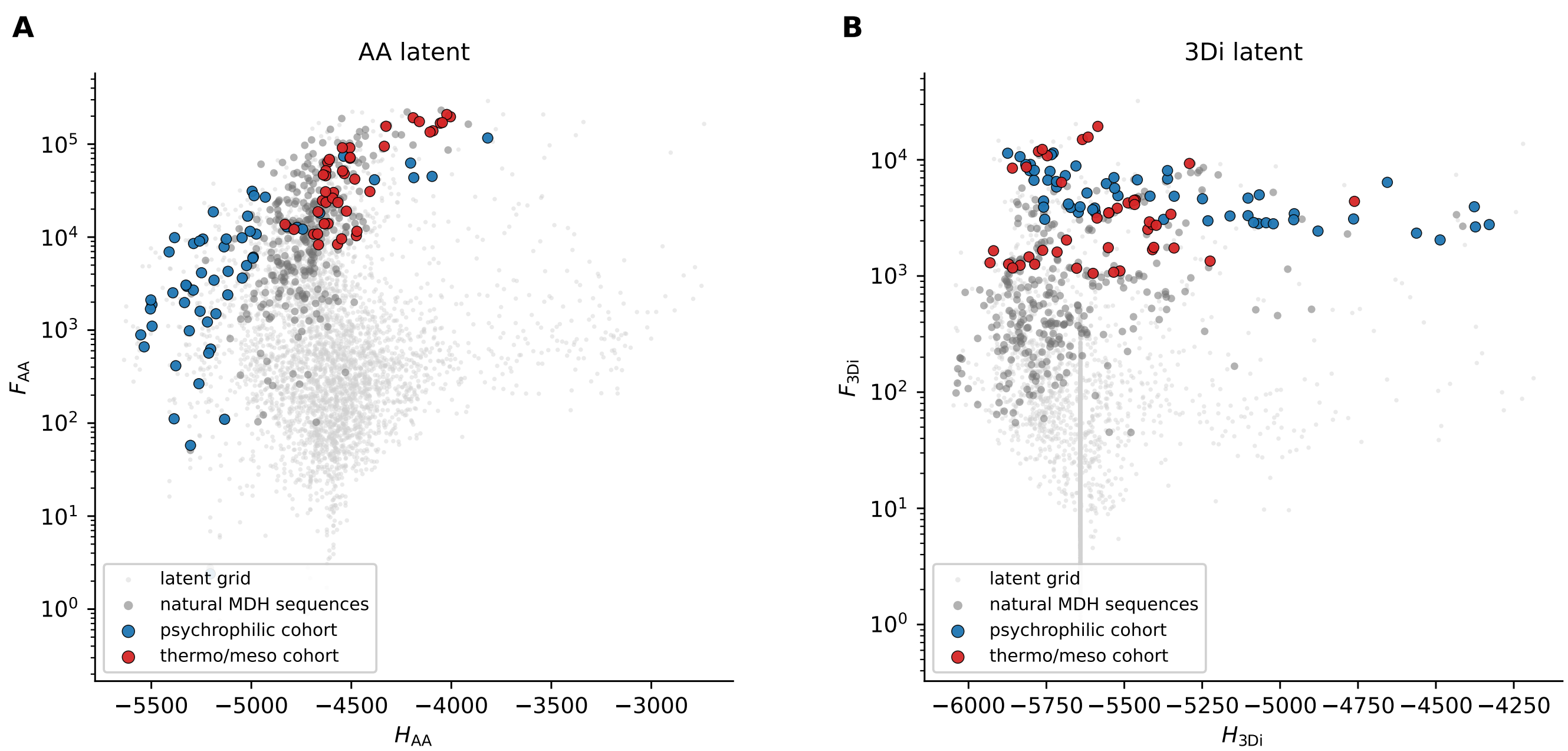


**Fig. S3.** Single-latent Fisher determinant against DCA Hamiltonian for MDH, shown for both AA and 3Di representations. Each panel plots latent points colored by category: latent-grid samples (light gray), natural MDH MSA sequences from the cached training set (medium gray), and the psychrophilic / thermo-mesophilic cohorts used in main-text Fig. 2. **(A)** AA latent: $F_{\mathrm{AA}}$ versus $H_{\mathrm{AA}}$. The two cohorts separate along the Hamiltonian axis, with psychrophilic at lower (more typical) $H_{\mathrm{AA}}$ and thermo/mesophilic at higher (more atypical) $H_{\mathrm{AA}}$; both occupy the high-Fisher region of the AA latent. **(B)** 3Di latent: $F_{\mathrm{3Di}}$ versus $H_{\mathrm{3Di}}$. The cohorts overlap on this plane, consistent with the structurally conserved nature of cytosolic MDHs across cohorts: when read through the structural-token Potts model, the two cohorts are nearly indistinguishable. These single-latent views are the geometric building blocks summarized by the AA-vs-3Di derivative axes of main-text Fig. 2D.

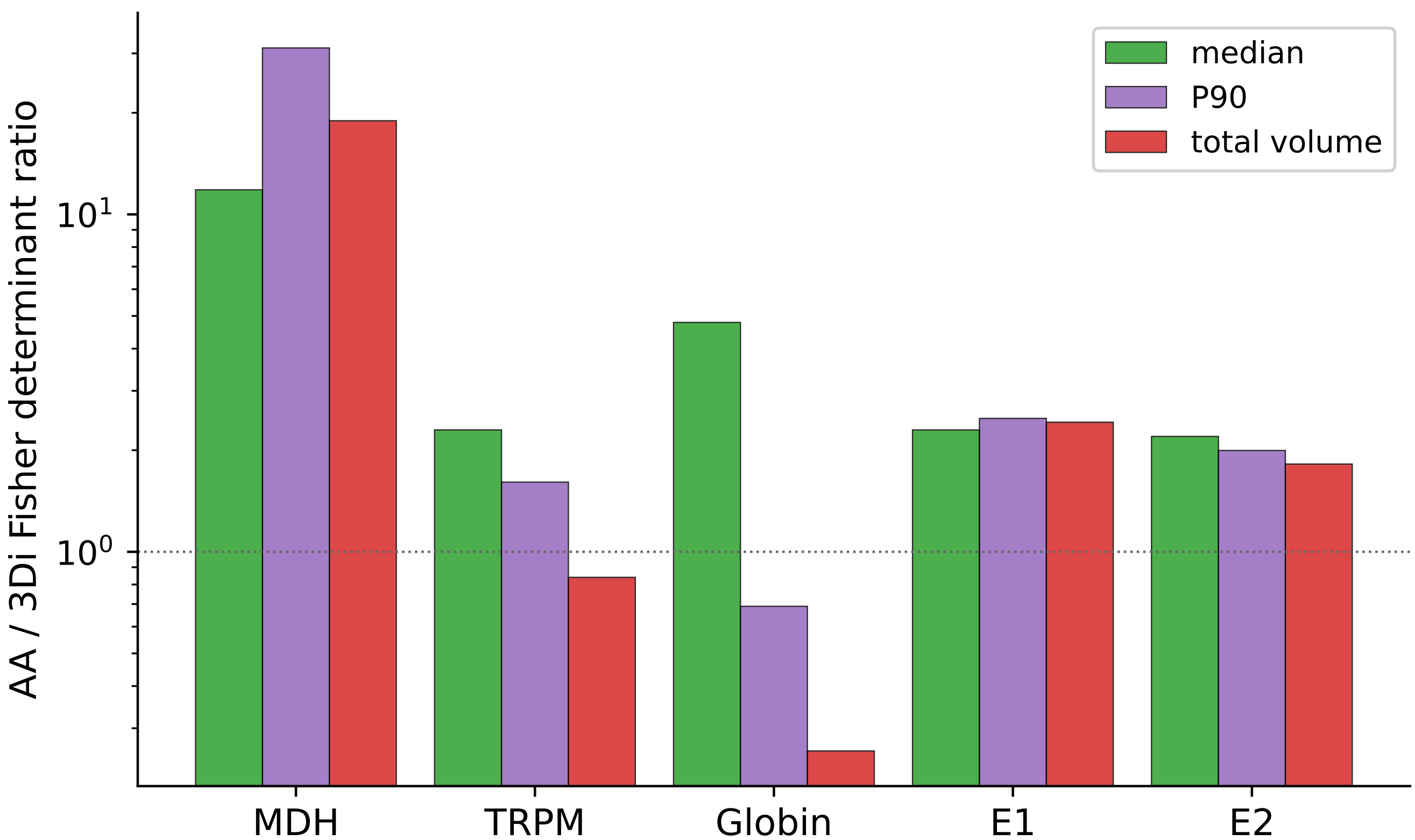


**Fig. S4.** Per-family Fisher determinant ratio between the AA and 3Di latents, summarised at three statistics on a logarithmic scale: median over the latent grid (green), ninetieth percentile (purple), and the integrated latent volume (red). Dashed horizontal line marks unity, at which the two decoders would have equal local sensitivity. MDH shows pronounced AA-over-3Di compression at all three summaries. Globin is strongly heterogeneous, so the two decoders dominate in different regions of the same landscape. TRPM and the Flaviviridae glycoproteins E1 and E2 all sit near parity with E1 and E2 approximately uniform across the three statistics and TRPM inverting mildly in the integrated volume. The compression is therefore a family-level descriptor rather than a universal constant, and it is independent of the consistent/complementary classification of Fig. 1D: that classification measures whether the two views order sequences the same way, whereas this ratio measures how much each decoder resolves per unit latent step. Because the tail and volume summaries are sensitive to grid regions in which the pullback metric is poorly conditioned, the median is the most robust of the three.

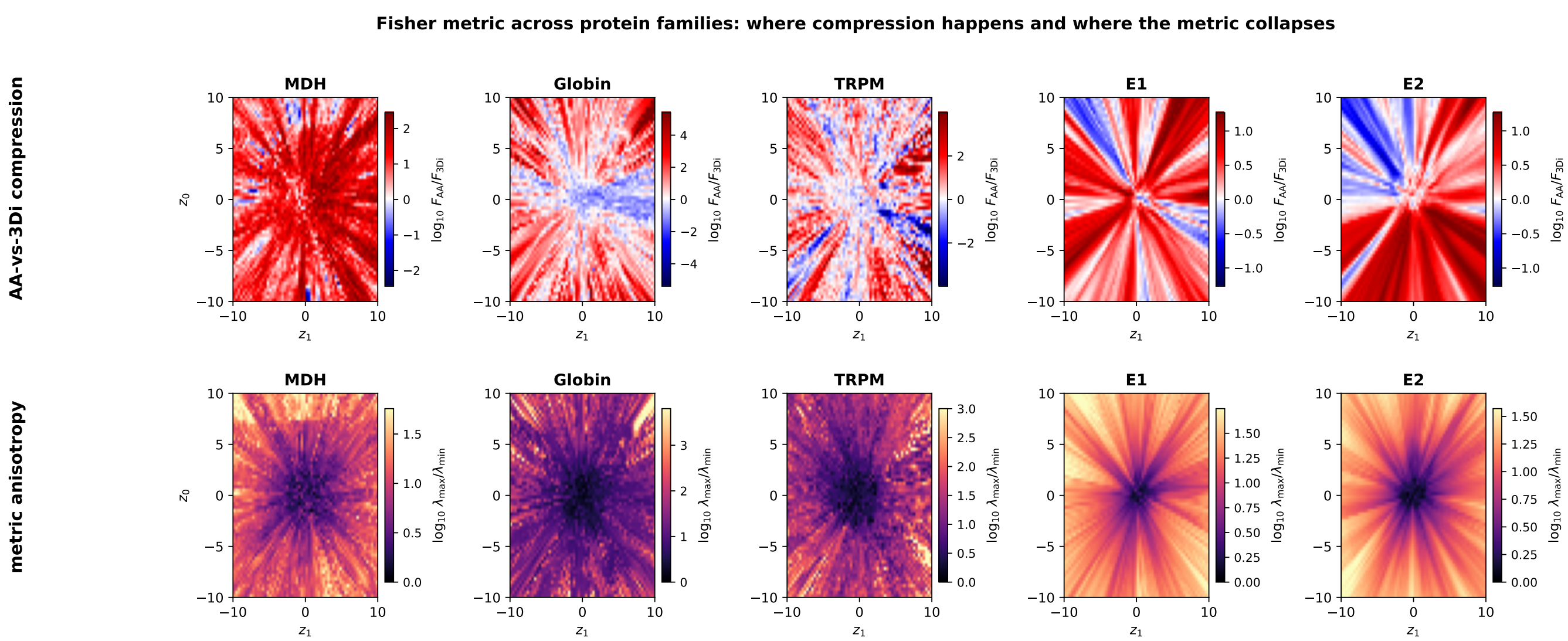


**Fig. S5.** Spatial Fisher-metric maps for five protein families: where each latent compresses, and where the local metric is anisotropic. **(Top row)** Logarithm of the AA-to-3Di Fisher determinant ratio across the latent grid of each family. Red regions are positions where the AA decoder is locally sharper (a small latent step gives a larger change in decoded sequence distribution under AA than under 3Di); blue regions are positions where the 3Di decoder is locally sharper. MDH is dominantly red across most of its latent, consistent with the family-wide compression reported in the main text; the Flaviviridae glycoproteins E1 and E2 are red-dominant but milder; globin and TRPM show mixed regions where the dominant representation flips. **(Bottom row)** Logarithm of the larger of the two Fisher-metric eigenvalue ratios at each latent point (the most anisotropic representation), measuring how strongly the local metric collapses toward a one-dimensional corridor. Globin and TRPM show pronounced anisotropic spokes corresponding to rank-deficient regions of their 3Di Fisher field, indicating that the structural latent has near-one-dimensional channels in these families; MDH, E1, and E2 are comparatively isotropic.

**Information-bottleneck framework: when are AA- and 3Di-aware representations complementary?**

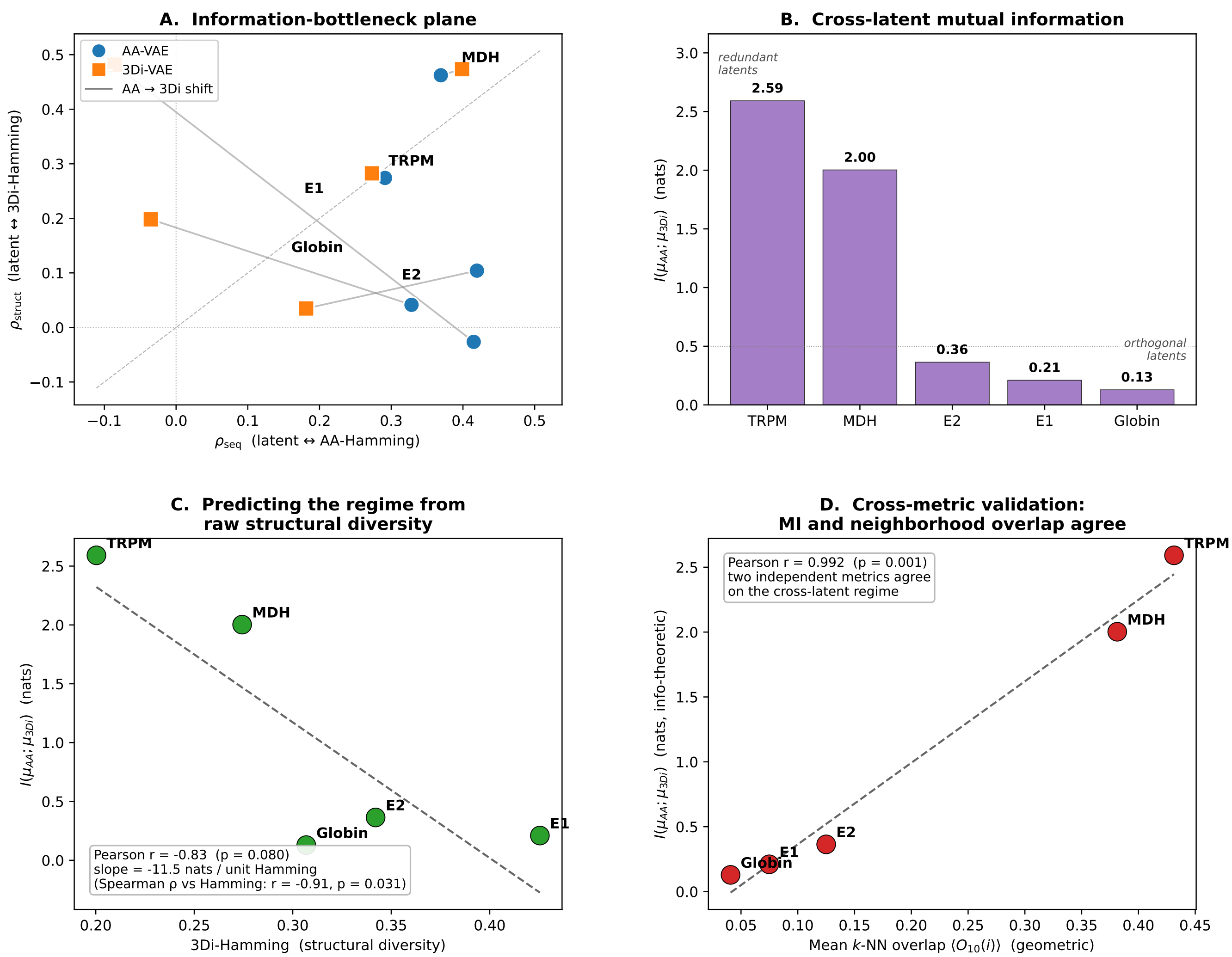


**Fig. S6.** Information-bottleneck framework for AA-aware and 3Di-aware latent representations across protein families. **(A)** Information-bottleneck plane. Each family is shown as two points (its AA-VAE and 3Di-VAE) at coordinates given by Spearman rank correlations of pairwise latent distance against pairwise AA-Hamming and pairwise 3Di-Hamming distances; gray lines connect the two representations of the same family. Families whose AA and 3Di points sit at similar coordinates carry redundant information across the two representations; families whose AA and 3Di points sit far apart on this plane carry orthogonal information. **(B)** Cross-latent mutual information between the AA and 3Di encoders for each family, estimated by a k-nearest-neighbour entropy method on paired training-sequence embeddings. Two regimes emerge cleanly: families with substantial mutual information across representations (TRPM, MDH) and families with mutual information close to zero (E2, E1, Globin). **(C)** Predictive law. The cross-latent mutual information from panel B is approximately linear in the family's mean pairwise 3Di-Hamming distance — a model-free measure of structural diversity read directly from the multiple sequence alignment, requiring no latent model. Low-diversity families produce redundant latents; high-diversity families produce orthogonal latents. **(D)** Cross-metric validation. The same regime classification is recovered by an entirely independent geometric metric, the mean k-nearest-neighbour overlap between AA and 3Di latent positions, which agrees with the information-theoretic mutual information across all five families.

 Divyanshu Shukla, Jonathan Martin, Faruck Morcos* and Davit A. Potoyan*

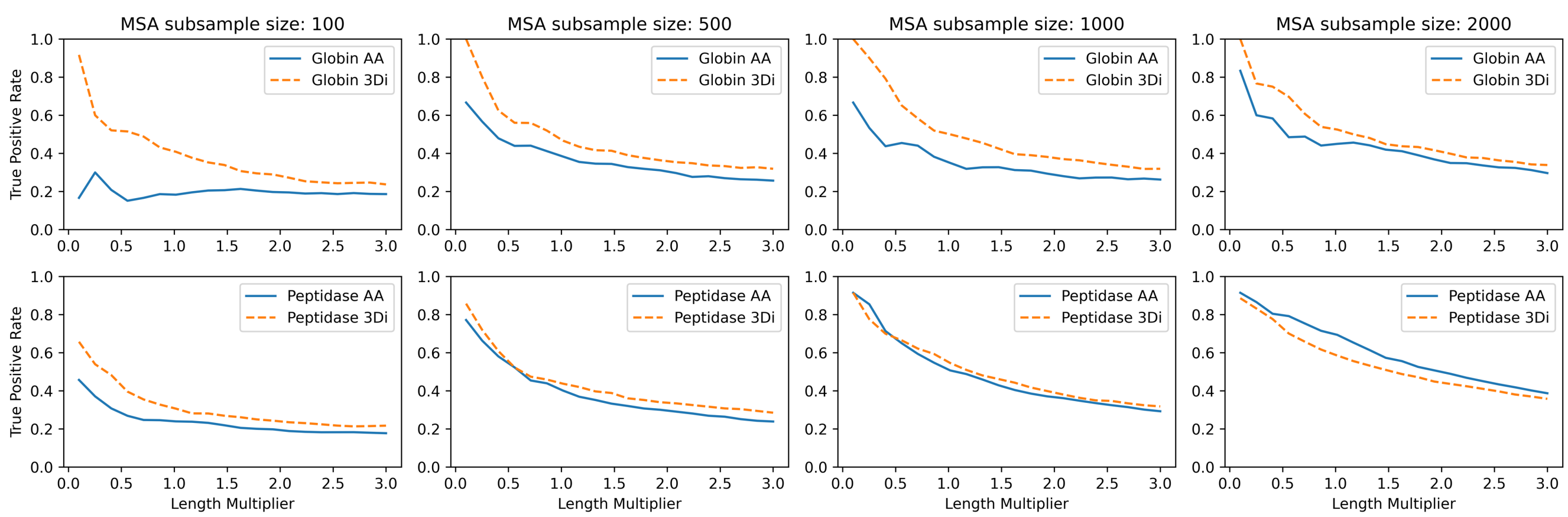


**Fig. S7.** Comparison of contact predictions using 3Di and amino-acid (AA) DCA across different MSA sizes. Precision of long-range contacts ($|i - j| > 4$) across top direct-information (DI) pairs ($0.1L$ to $3L$) against reference structures (Globin PDB: 1xz2; Peptidase PDB: 1AEC). Each column represents an MSA subsampled from the corresponding full alignment; rows compare AA-based and 3Di-based DCA. The crossover behavior with MSA depth supports the bias–variance interpretation in the main text: 3Di provides cleaner contacts at modest sequence depth, while the AA alphabet eventually surpasses it given enough sequences.

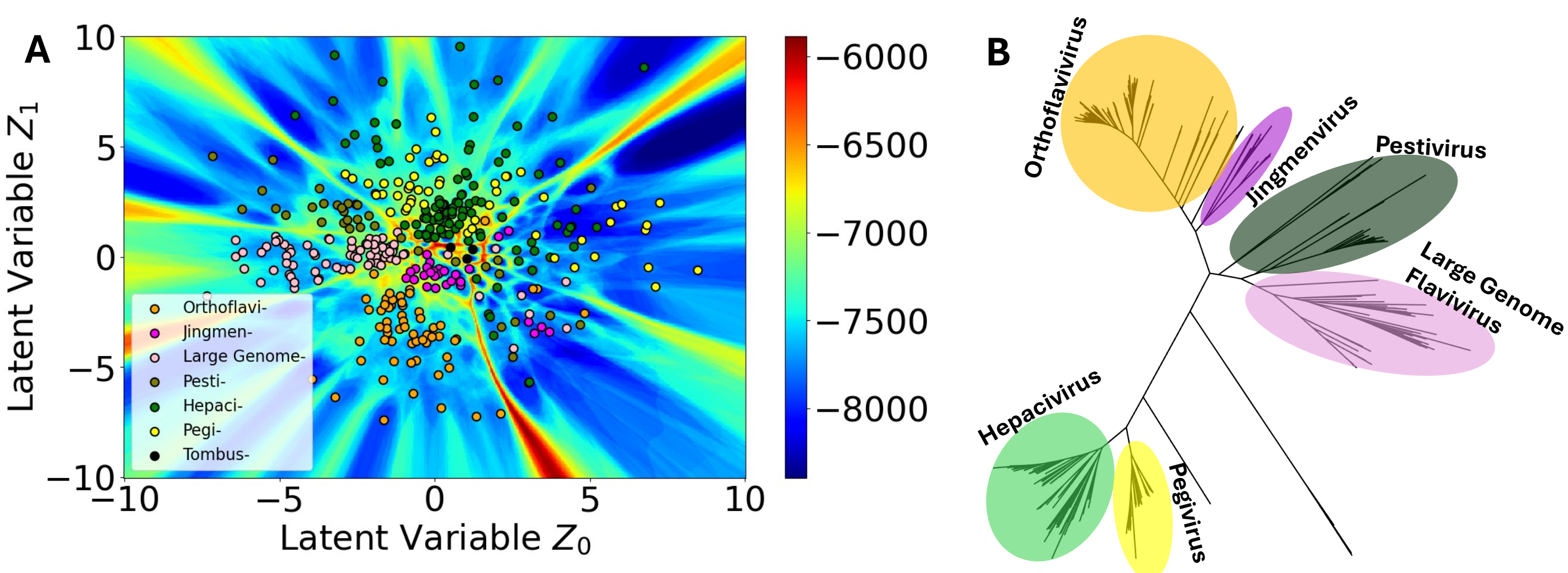


**Fig. S8.** Landscape and phylogeny of Flaviviridae from the conserved NS5 (RdRp) region. **(A)** Latent space of Flaviviridae 3Di sequences encoded from the highly conserved NS5 region, colored by genera. **(B)** Phylogenetic reconstruction showing the placement of orthoflaviviruses, jingmenviruses, pestiviruses, large-genome flaviviruses, hepaciviruses, and pegiviruses. The genera grouping in the latent space is consistent with the topology of the RdRp-based tree, providing a positive control that 3Di latents recover known evolutionary structure for a conserved locus.

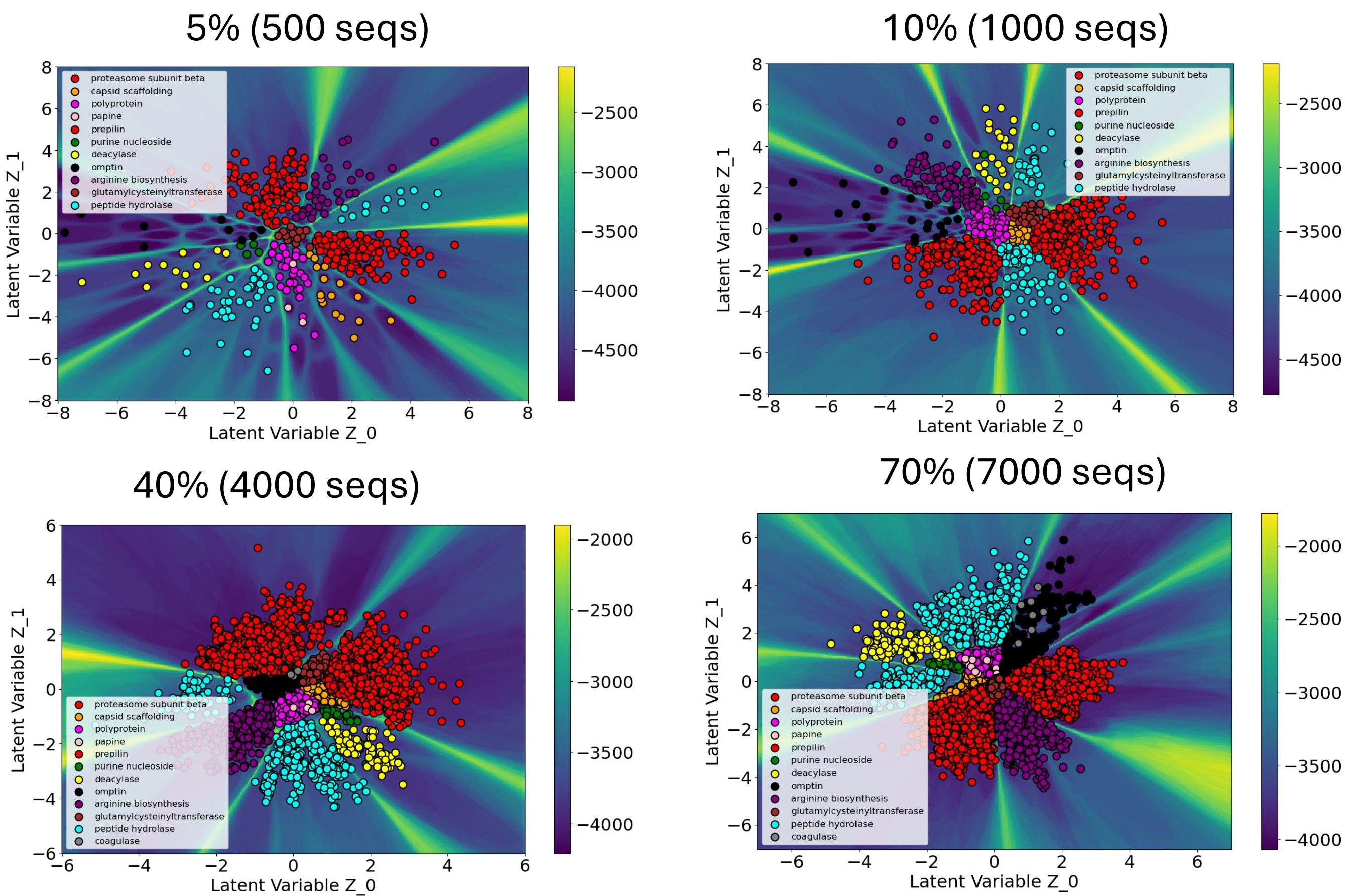


**Fig. S9.** Effect of MSA sample size on the VAE latent landscape and Potts Hamiltonian surface. Latent generative landscapes constructed from subsampled peptidase MSAs containing 5%, 10%, 40%, and 70% of the full sequence dataset. Colored points represent training sequences annotated by functional class; the background surface shows the DCA/Potts Hamiltonian projected onto the 2D VAE latent space. Across all sampling fractions, the VAE preserves the same coarse family-level cluster organization. In contrast, the fine-scale shape of the Hamiltonian surface depends on sampling depth: at low counts (5–10%) the landscape contains more narrow local minima, whereas at higher fractions (40–70%) the surface becomes smoother and basin-level structure dominates. Thus the basin- and cluster-level organization is robust to sample size, while the granularity of local minima reflects sampling density.

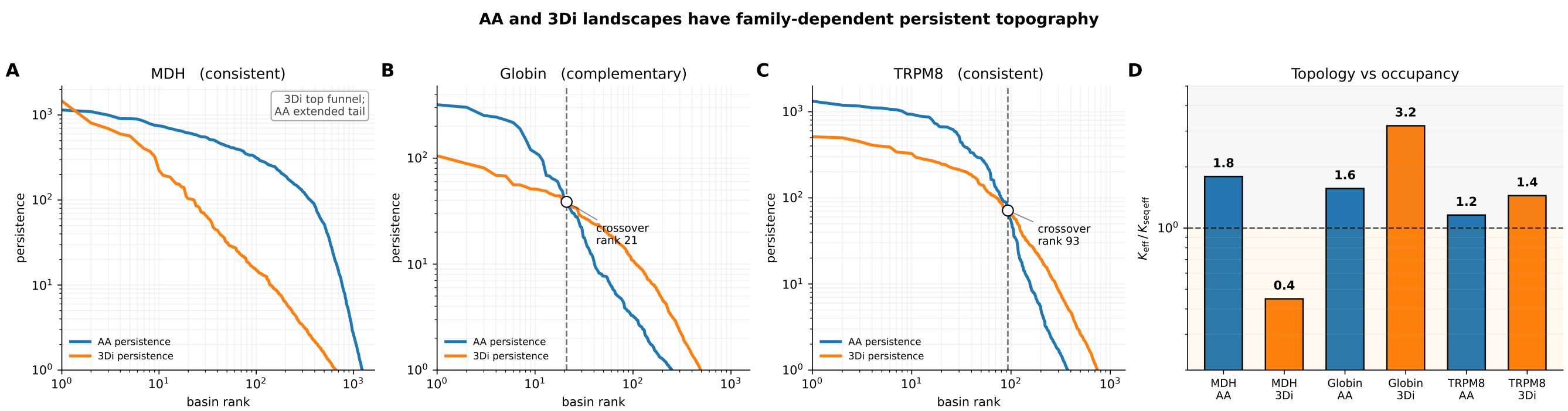


**Fig. S10.** AA-VAE and 3Di-VAE landscape topography across three protein families. **(A–C)** Basin persistence in the DCA Hamiltonian landscape, ranked from the deepest to the shallowest basin, for the sequence-aware (AA-VAE, blue) and structure-aware (3Di-VAE, orange) representations of MDH (A), globin (B), and TRPM8 (C). Basins are obtained without hand-selection: the $500 \times 500$ smoothed Hamiltonian grid is watershed-segmented from all detected local minima, and the persistence of each basin is the height interval $p_b = s_b - H_b^{\min}$ between its minimum and the saddle at which it merges into a deeper basin under a sublevel-set filtration (see "Definitions of quantitative measures"). Persistence is stable to perturbations of the underlying surface; long-persistence basins are robust topographic features while short-persistence ones are finite-sampling noise. For globin and TRPM8 the two curves cross at a well-defined rank, beyond which the structure-aware landscape contains more fine-scale basins than the sequence-aware one, consistent with an interpretation of AA persistence as reflecting phylogenetic depth and 3Di persistence as reflecting conformational breadth. For MDH the sequence-aware representation dominates the ladder throughout, with the structure-aware landscape compressed into a few dominant funnels. **(D)** Ratio of the effective topological basin count $K_{\mathrm{eff}}$ to the effective sequence-occupancy basin count $K_{\mathrm{seq\,eff}}$, for each of the six landscapes. Both are participation ratios (effective counts), $K_{\mathrm{eff}} = (\sum_b p_b)^2 / \sum_b p_b^2$ computed from the basin persistences and $K_{\mathrm{seq\,eff}}$ from the per-basin training-sequence counts; the participation ratio equals the true number of basins when all are equal and falls toward one when a single basin dominates. Bars above the dashed reference line indicate landscapes whose persistence diagrams predict more basins than training sequences actually populate; the MDH structure-aware landscape is the single inversion, where training sequences spread across more basins than persistence-based filtering alone identifies, consistent with the structural-funnel compression visible in panel A.

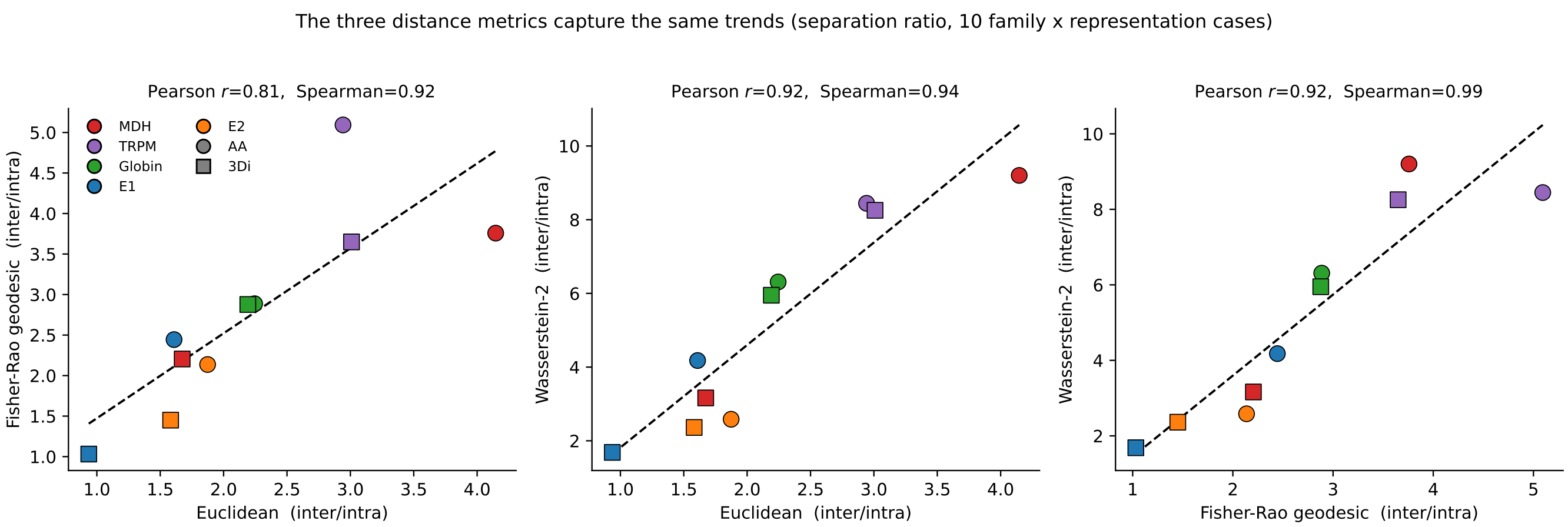


**Fig. S11.** The three latent-distance metrics capture the same trends, so distance-based conclusions are robust to the choice of metric. For each of the five families, in both the AA-VAE and 3Di-VAE latents (ten cases in total), we computed the inter-basin to intra-basin separation ratio of the biologically labeled groups under three distance metrics: Euclidean latent distance, energy-minimized Fisher-Rao geodesic distance, and Wasserstein-2 distance between group distributions. The three panels show the pairwise relationships across the ten cases (markers colored by family; circles, AA latent; squares, 3Di latent; dashed line, linear fit). The metrics are strongly correlated (Pearson $r = 0.81$ to $0.92$; Spearman $\rho = 0.92$ to $0.99$), with the Fisher-Rao geodesic and Wasserstein-2 rankings nearly identical ($\rho = 0.99$). Which cases separate strongly versus weakly is therefore essentially independent of the distance metric used.

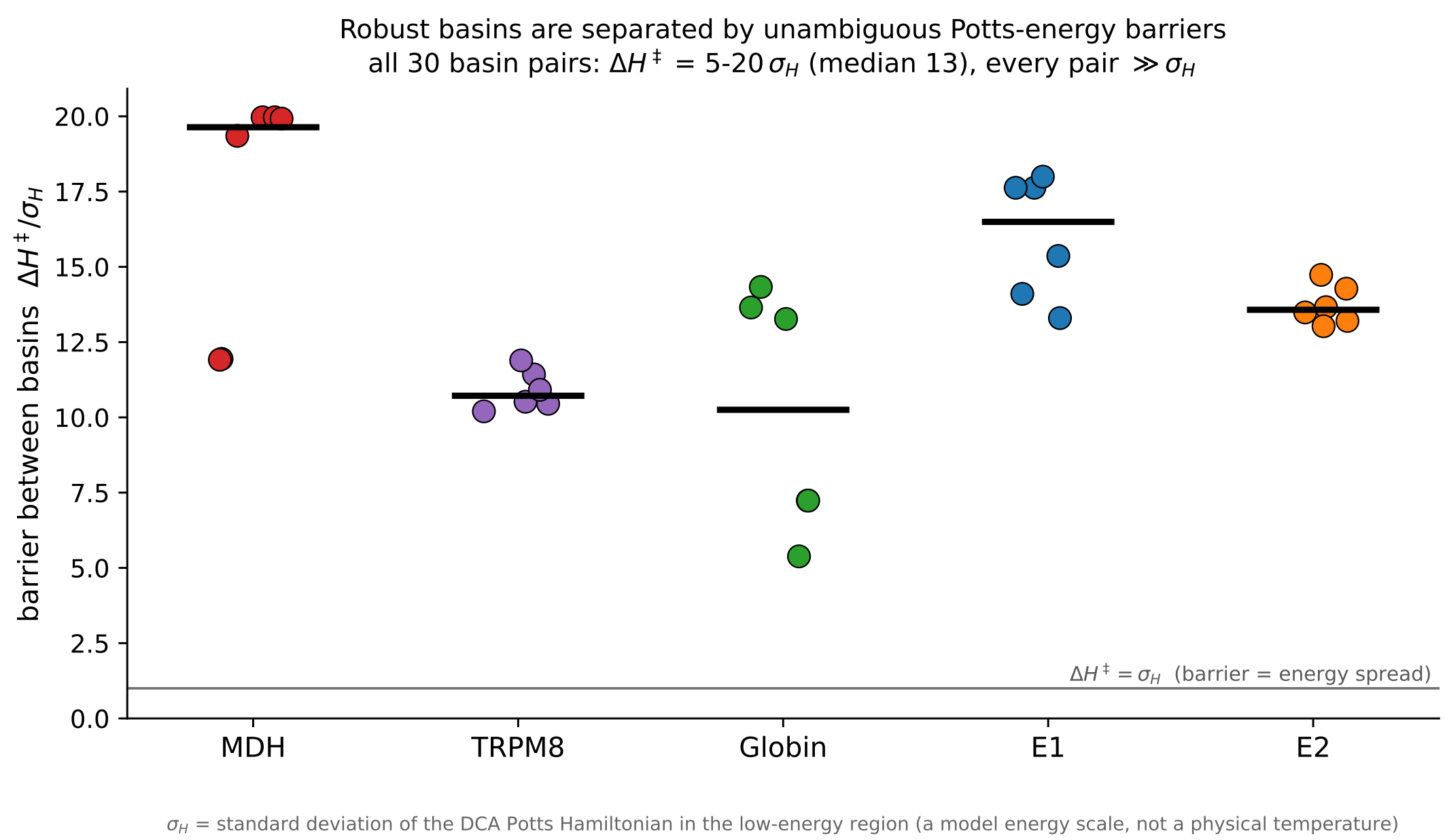


**Fig. S12.** Robust basins are separated by unambiguous Potts-energy barriers, independent of any distance metric. For each of five families, the top four robust basins of the 3Di-VAE Hamiltonian landscape were identified by watershed on the smoothed surface, and the barrier between every pair of basins was computed as the minimax (lowest saddle) along the minimum-energy path. Each point is one basin pair; the horizontal bar is the per-family median. Barriers are expressed in units of $\sigma_H$, the standard deviation of the DCA Potts Hamiltonian in the low-energy region of the landscape, which is a model energy scale and not a physical temperature (distinct from the natural-sequence spread $\sigma_{H,\mathrm{nat}}$ used for the Langevin barriers of Fig S16; see "Definitions of quantitative measures"). All 30 basin pairs are separated by barriers of $5$ to $20\,\sigma_H$ (median $13\,\sigma_H$), far above the $\Delta H^\ddagger = \sigma_H$ reference; the landscape therefore partitions sequences into energetically distinct basins as a topological property of the energy surface, with no appeal to a latent distance. This is the metric-free counterpart of Supplementary Fig. S11, and the distilled, distance-independent statement of the cohort-barrier analyses in Supplementary Fig. S16.

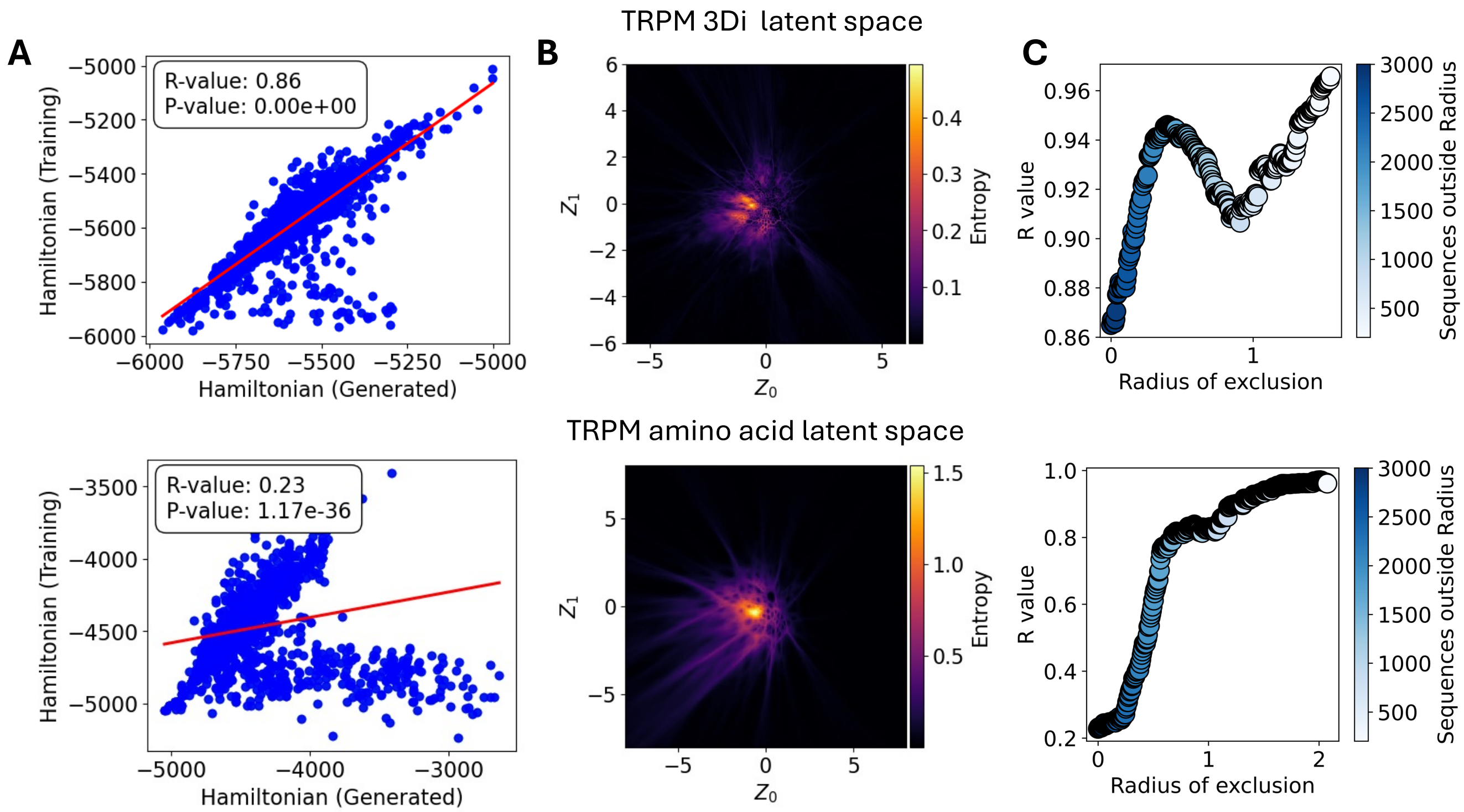


**Fig. S13.** Decoder performance and entropy analysis for the TRPM family. **(A)** Comparison of DCA-Hamiltonian scores between input training sequences and sequences generated at the corresponding encoded $\mu$-coordinate, in both the 3Di (top) and amino-acid (bottom) latent spaces. The 3Di latent yields a substantially higher input-vs-decoded correlation ($R = 0.86$) than the AA latent ($R = 0.23$). **(B)** Entropy of the decoder's softmax distribution at each latent coordinate. The 3Di latent shows lower and more spatially localized entropy compared with the broader, higher-entropy AA latent. **(C)** Correlation between input and generated Hamiltonians as a function of radial exclusion from the high-entropy latent-space centre. Excluding central, high-uncertainty sequences improves the correlation in both landscapes, with the 3Di space achieving higher correlation even near the centre. Color bar indicates the number of sequences remaining after each exclusion step. We note that this TRPM-specific pattern does not generalise universally across families: in cross-family checks the decoder-Hamiltonian correlation is family-dependent (e.g., for MDH the ordering inverts, with $R \approx 0.91$ on the AA latent and $R \approx 0.14$ on the 3Di latent), while per-position decoder accuracy remains comparably high ($\sim 93$–$95\%$) on both latents. The principled, parameterization-invariant version of the AA-vs-3Di decoder comparison lives in the Fisher determinant (Supplementary Fig. S4) and the Fisher-Rao geodesic (Supplementary Fig. S14), which behave more uniformly across families.

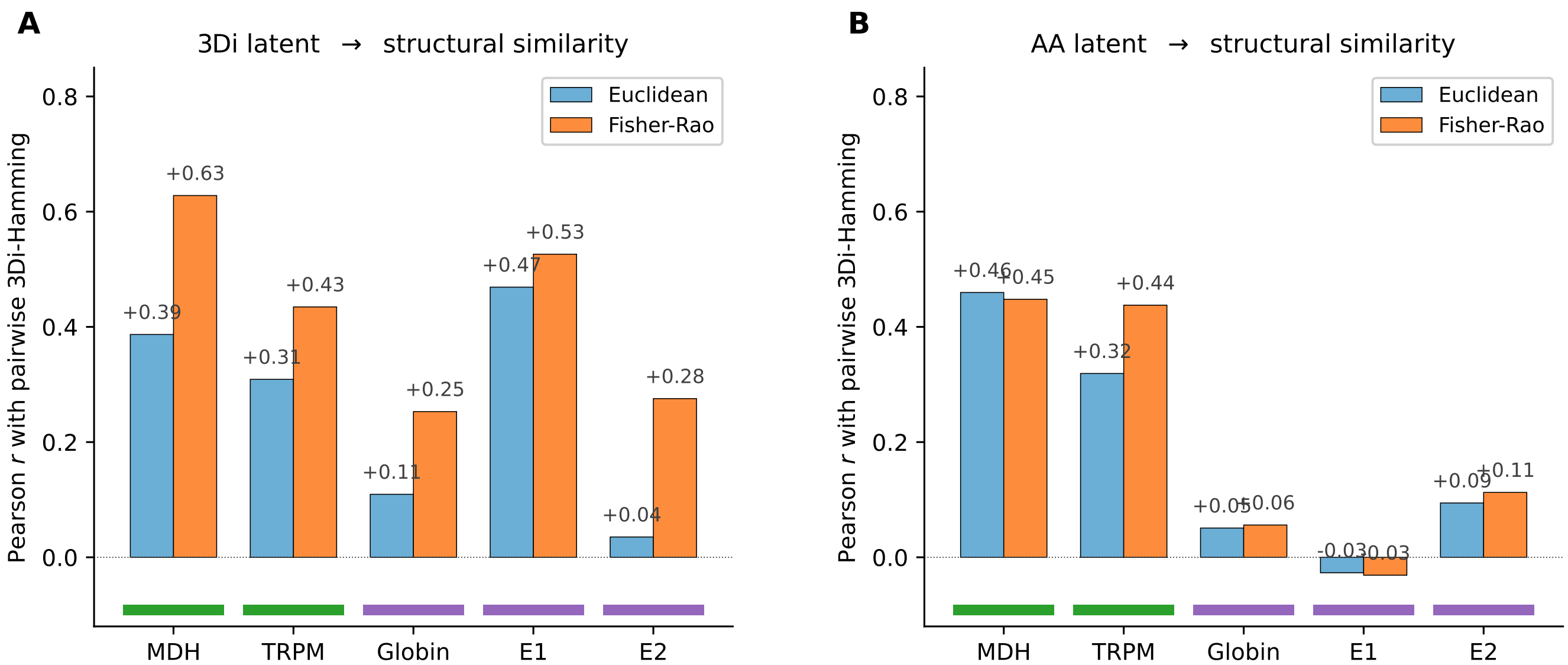


**Fig. S14.** Recovery of pairwise structural similarity from the 2D latents. Each panel correlates a latent-space distance with pairwise 3Di-Hamming computed on the same pair of sequences; Euclidean distance (blue) is compared with the Fisher-Rao geodesic length obtained by integrating the Fisher line element along the connecting segment (orange). The Fisher information metric on the latent is constructed as in Arvanitidis et al.(2) and detailed in the Methods. Colored bars below each family indicate its regime from main text Fig. 1D (green = consistent, purple = complementary). **(A)** 3Di latent. The 3Di latent recovers structural similarity in every family; with the Fisher-Rao metric the correlation improves in all five families, often substantially. This is the principled distance to use on the 2D latent. **(B)** AA latent. The AA latent recovers structural similarity only for the consistent-regime families (MDH, TRPM), where AA and 3Di Hamming co-vary. For the complementary-regime families (Globin, E1, E2) the AA latent is essentially uninformative about structural similarity (E1 is even slightly negative), and the Fisher-Rao improvement adds nothing because there is no underlying signal to refine. Together these two panels make the regime-dependence explicit: structural similarity is read off the 3Di latent universally, while reading it off the AA latent requires the family to lie in the consistent regime. To verify that this geometric improvement is not an artifact of using a 3Di-derived reference, we additionally replace pairwise 3Di-Hamming with pairwise TM-score (computed from AlphaFold structures and independent of the 3Di alphabet) for the MDH cohort and for the globin AlphaFold structure set; the Fisher-Rao geodesic still recovers structural similarity better than naive Euclidean distance on the 3Di latent in both cases, with the strongest correlation recovered for globin where TM-score spans the widest dynamic range across functional sub-families.

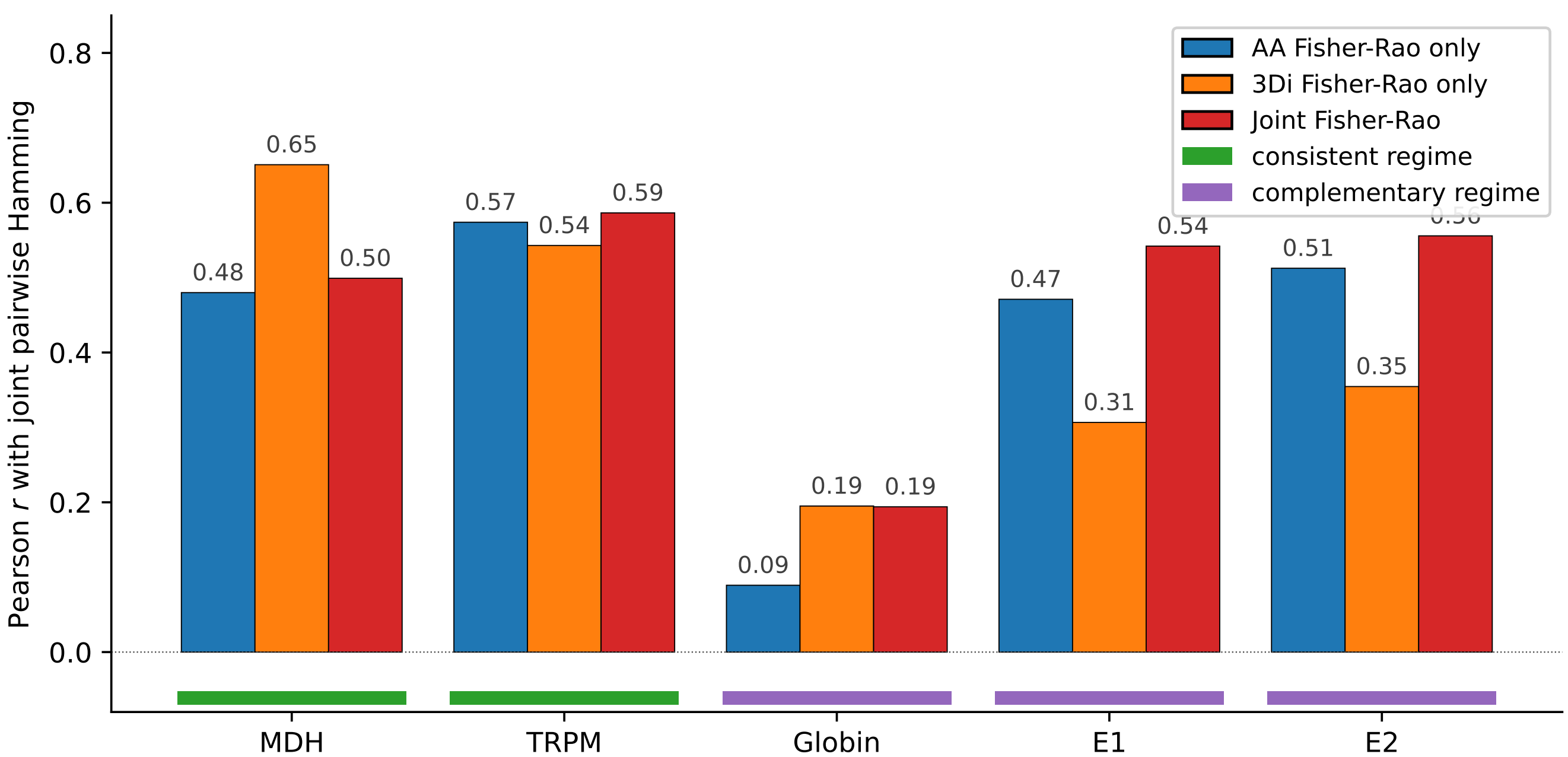


**Fig. S15.** Joint AA + 3Di Fisher-Rao distance is the strongest descriptor of pairwise similarity in the complementary regime. For each family, three Fisher-Rao distances between training-sequence pairs (matched between AA and 3Di) are correlated with the joint pairwise Hamming distance: AA Fisher-Rao alone (blue), 3Di Fisher-Rao alone (orange), and the joint Fisher-Rao $\sqrt{d_{\mathrm{AA}}^2 + d_{\mathrm{3Di}}^2}$ (red). The colored bar below each family indicates its regime from Fig. 1D (green = consistent, purple = complementary). For *consistent*-regime families (MDH, TRPM), one representation suffices and the joint distance is comparable. For *complementary*-regime families (Globin, E1, E2), the joint Fisher-Rao distance outperforms either single representation, confirming that AA and 3Di carry distinct, complementary information that combines productively. This validates the framework's recommendation to interpret families jointly via Fisher-Rao when AA and 3Di latents are complementary.

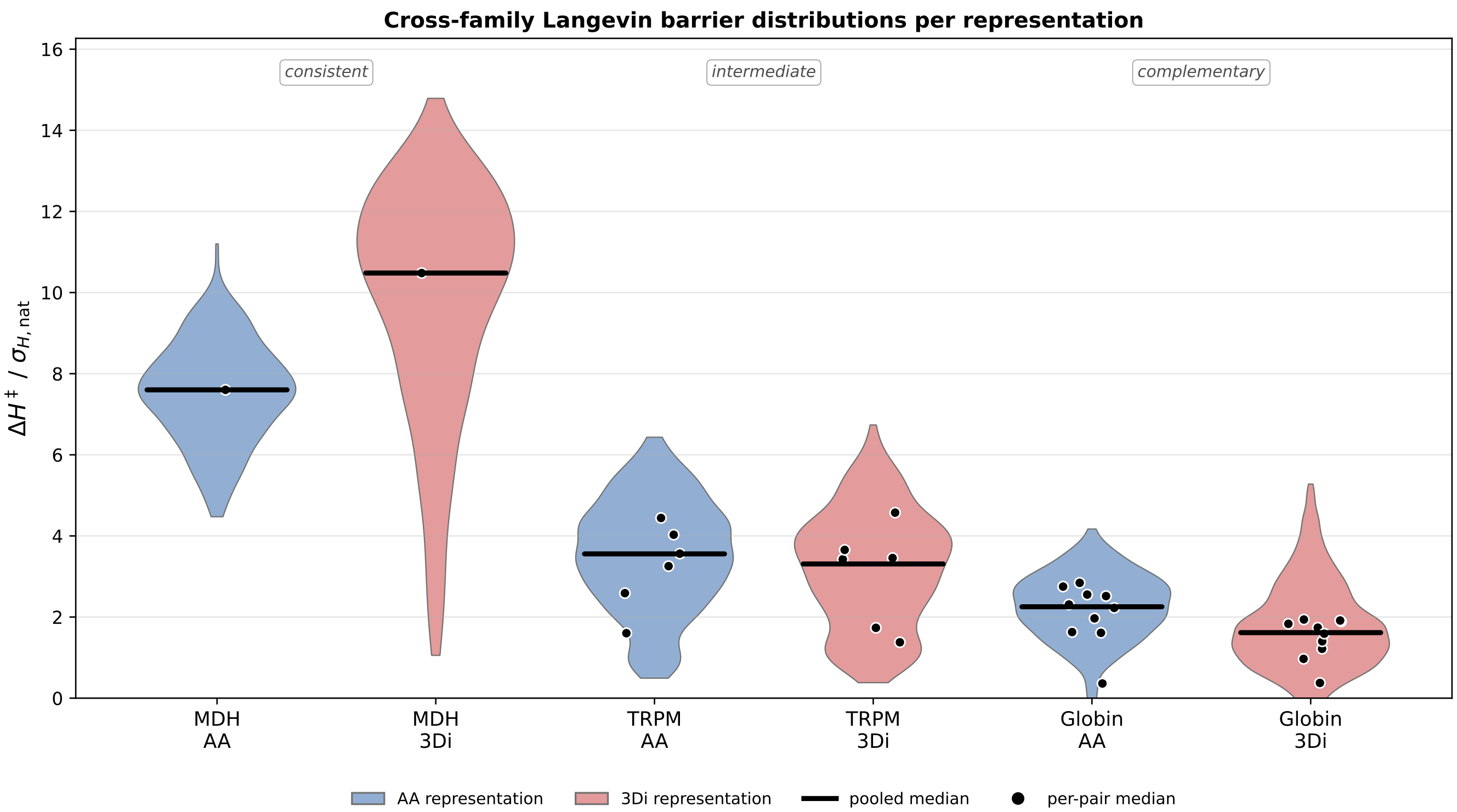


**Fig. S16.** Cross-family distributions of Langevin cohort-to-cohort barriers per representation. Violins show pooled per-trajectory barrier heights aggregated across all cohort-pair Langevin runs per family and representation (one binary pair for MDH, six pairs for TRPM, ten pairs for Globin), in units of each family's natural-sequence Hamiltonian spread $\sigma_{H,\mathrm{nat}}$ (the standard deviation of the Potts Hamiltonian at the latent positions of the natural training sequences; see "Definitions of quantitative measures"). Each per-trajectory barrier is the maximum smoothed Hamiltonian reached along the productive crossing segment minus the source-basin floor. Thick horizontal lines mark the pooled median; black dots overlay each cohort pair's individual median. Families are ordered along the regime axis from consistent (MDH, deepest barriers) to complementary (Globin, shallowest barriers); AA and 3Di representations are shown in blue and red, respectively. The 3Di representation amplifies cohort-to-cohort barriers in the consistent regime, leaves them essentially unchanged for the intermediate TRPM, and softens them in the complementary regime, mirroring the regime-dependent compression patterns reported by the Fisher-information analysis.

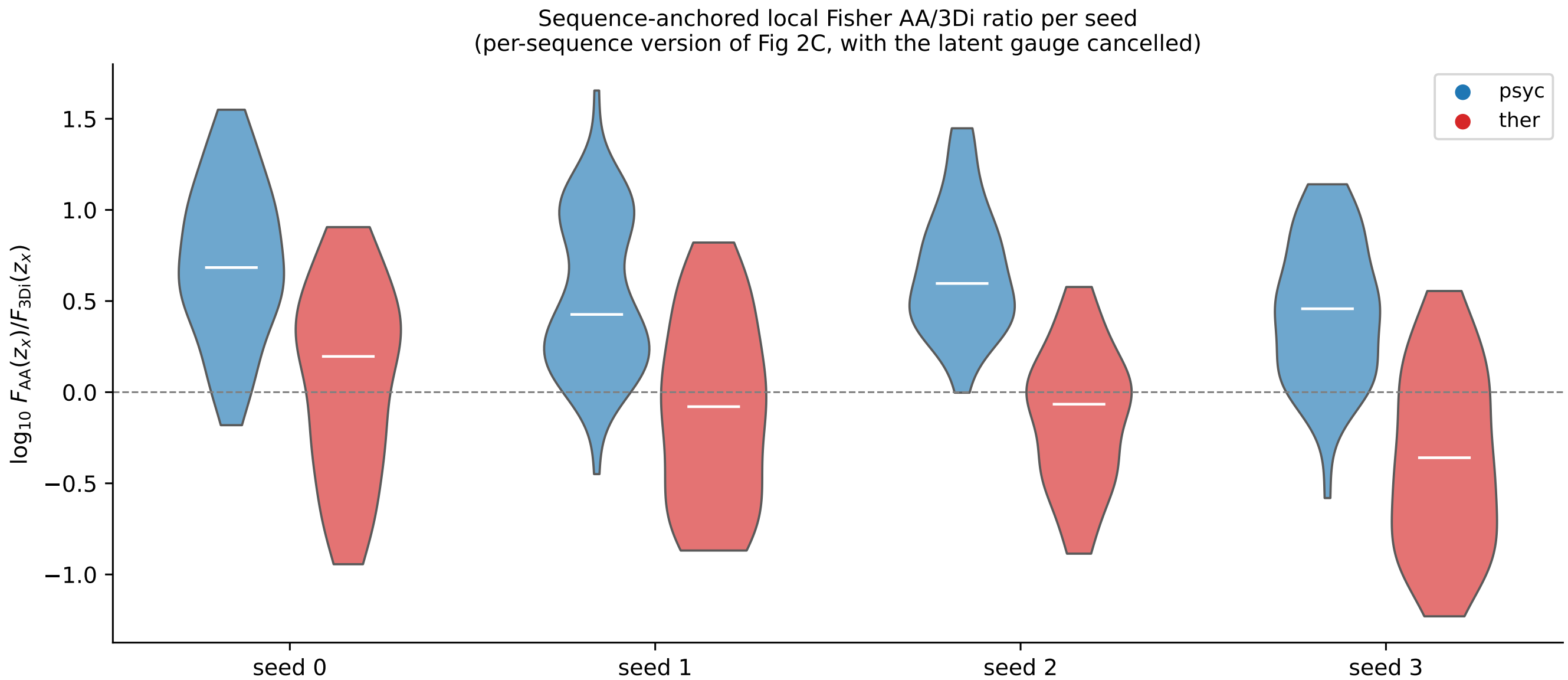


**Fig. S17.** Sequence-anchored local Fisher AA/3Di ratio per seed, the sequence-level version of main-text Fig. 2C with the latent gauge cancelled. For each of the four random seeds, the cohort sequences are encoded under both the AA-VAE and the 3Di-VAE of that seed; the pullback Fisher determinants are then evaluated at each sequence's encoded latent location, and the violins show the per-sequence distribution of $\log_{10}\big(F_{AA}(z_x)/F_{3Di}(z_x)\big)$ for the psychrophilic (blue) and thermo/mesophilic (red) cohorts. The median for the psychrophilic cohort is reproducibly positive across all four seeds ($+0.68$, $+0.43$, $+0.60$, $+0.46$ in $\log_{10}$ units; mean $+0.54 \pm 0.12$): the AA decoder is consistently locally sharper at the psychrophilic sequence locations. The median for the thermo/mesophilic cohort spans a range of $0.56$ dex across seeds, from $+0.20$ in seed $0$ to $-0.36$ in seed $3$, so the sign of the local AA-versus-3Di ratio at this cohort is not strictly seed-robust. Together with Supplementary Fig. S2, this clarifies that the seed-robust load-bearing quantity in the AA-versus-3Di compression story is the within-versus-between cohort contrast at the latent-distance level, not the per-pixel or per-sequence local Fisher value. Underlying script: `76_seed_local_fisher_ratio.py`.

## References


1. A Kraskov, H Stögbauer, P Grassberger, Estimating mutual information. *Phys. Rev. E* **69**, 066138 (2004).
2. G Arvanitidis, M González-Duque, A Pouplin, D Kalatzis, S Hauberg, Pulling back information geometry in *Proceedings of The 25th International Conference on Artificial Intelligence and Statistics (AISTATS)*, Proceedings of Machine Learning Research, eds. G Camps-Valls, FJR Ruiz, I Valera. (PMLR), Vol. 151, pp. 4872–4894 (2022).
3. S Cocco, R Monasson, F Zamponi, *From Statistical Physics to Data-Driven Modelling: with Applications to Quantitative Biology.* (Oxford University Press), (2022).
4. DA Sivak, GE Crooks, Thermodynamic metrics and optimal paths. *Phys. Rev. Lett.* **108**, 190602 (2012).
